# Controllable Assembly of Upconversion Nanoparticles Enhanced Tumor Cell Penetration and Killing Efficiency

**DOI:** 10.1002/advs.202001831

**Published:** 2020-11-07

**Authors:** Zhen Zhang, Juwita Norasmara Rahmat, Ratha Mahendran, Yong Zhang

**Affiliations:** ^1^ Department of Biomedical Engineering Faculty of Engineering National University of Singapore Singapore 117583 Singapore; ^2^ Department of Surgery Yong Loo Lin School of Medicine National University of Singapore Singapore 119228 Singapore; ^3^ NUS Graduate School for Integrative Sciences and Engineering National University of Singapore Singapore 117456 Singapore

**Keywords:** clusters, nanoparticle assembly, photodynamic therapy, tumor cell penetration, upconversion

## Abstract

The use of upconversion nanoparticles (UCNPs) for treating deep‐seated cancers and large tumors has recently been gaining momentum. Conventional approaches for loading photosensitizers (PS) to UCNPs using noncovalent physical adsorption and covalent conjugation had been previously described. However, these methods are time‐consuming and require extra modification steps. Incorporating PS loading during the controlled UCNPs assembly process is seldom reported. In this study, an amphiphilic copolymer, poly(styrene‐*co*‐maleic anhydride), is used to instruct UCNPs assembly formations into well‐controlled UCNPs clusters of various sizes, and the gap zones formed between individual UCNPs can be used to encapsulate PS. This nanostructure production process results in a considerably simpler and reliable method to load PS and other compounds. Also, after considering factors such as PS loading quantity, penetration in 3D bladder tumor organoids, and singlet oxygen production, the small UCNPs clusters displayed superior cell killing efficacy compared to single and big sized clusters. Therefore, these UCNPs clusters with different sizes could facilitate a clear and deep understanding of nanoparticle‐based delivery platform systems for cell killing and may pave a new way for other fields of UCNPs based applications.

## Graphical abstract

Controlled formation of upconversion nanoparticles (UCNPs) clusters of various sizes (individual; dimer and trimer; small and big clusters) is achieved via regulation of poly(styrene‐*co*‐maleic anhydride) polymer to UCNPs weight ratio used during the assembly process. The small‐sized UCNPs‐Ce6 clusters (60 nm) are the best performing nanocarrier when compared to single and big UCNPs‐Ce6 clusters, displaying enhanced tumor cell killing efficacy.

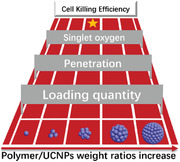

## 1 Introduction

Photodynamic therapy (PDT) is a minimally invasive therapeutic procedure and is a viable alternative approach for the treatment of various dermatological conditions such as precancerous lesions, acne, and cutaneous infections.^[^ ^1^ ^]^ PDT applications in early‐stage malignancies of the skin, bladder, esophagus, head, and neck demonstrated favorable clinical response rates, low toxicity, and remarkable aesthetic outcomes.^[^ ^2^, ^3^, ^4^ ^]^ However, the use of PDT to treat larger and deep‐seated tumors has yet to gain traction. Their unpopularity is due to the visible light source used, which is absorbed by endogenous chromophores,^[^ ^5^ ^]^ and has poor penetration in healthy and diseased tissues.^[^ ^6^ ^]^ Visible light also undergoes scattering in tissues because of biological components such as fat, blood, and melanosomes.^[^ ^7^ ^]^ The use of near‐infrared (NIR) light can overcome the abovementioned challenges as NIR light can relieve the scattering effect and has better tissue penetration. However, most photosensitizers (PS) are excited by light in the UV–visible (UV–vis) light range.^[^ ^8^ ^]^ To overcome this bottleneck, upconversion nanoparticles (UCNPs) used for PDT applications have received widespread attention in recent years. Under NIR light excitation, UCNPs can emit high‐energy UV–vis light, which will then activate surrounding PS molecules to produce singlet oxygen (^1^O_2_) and kill cancerous cells.

**Figure 1:**
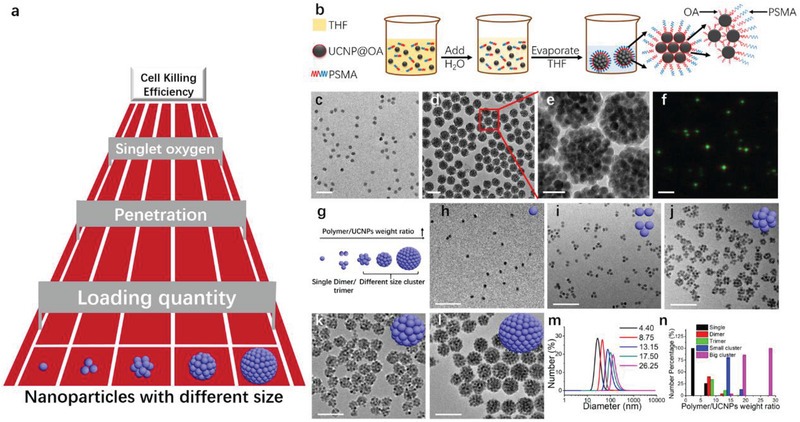
Polymer induced UCNPs self‐assembly. a) A schematic of factors influencing the tumor cell killing efficacy when using UCNPs of different sizes. b) Schematic of PSMA induced self‐assembly of single UCNPs into UCNPs clusters. c) TEM image of UCNPs (NaYF_4_: 20% Yb, 2% Er, the diameter is about 22 nm, green fluorescence). d) TEM image and e) magnified TEM image of UCNPs sphere clusters. f) Luminescence microscopy image of spherical UCNPs clusters under 980 nm excitation. Scale bar, 200 nm for (c,d); 50 nm for (e) and 2 µm for (f). g) Schematic representation of controllable UCNPs clusters morphology with different polymer/UCNPs weight ratios. Representative TEM micrographs of UCNPs morphology in water after THF evaporation with different polymer/UCNPs weight ratios: h) 4.40; i) 8.75; j) 13.15, k) 17.50; l) 26.25. Scale bar, 200 nm. m) DLS results of typical UCNPs morphology in water after THF evaporation with different polymer/UCNPs weight ratio. n) Statistical analysis of fractions of different particles for different polymer/UCNPs weight ratios. At least 100 objects were analyzed for each sample.

The light source is not the only challenge preventing the promise of PDT to be fully realized. PDT success depends on two other vital factors that must occur in parallel within the targeted tumor mass; selective accumulation and adequate penetration of administered PS. PDT for cancer therapy requires the accumulation of PS in diseased tissues, which will then be excited to generate enough ^1^O_2_ to induce cell death.^[^ ^9^, ^10^ ^]^ There are two commonly used approaches to load PS molecules onto UCNPs to date: non‐covalent physical adsorption and covalent conjugation via chemical linking.^[^ ^11^ ^]^ The most widely utilized adsorption strategy is silica encapsulation. ^[^ ^12^ ^]^ The fabrication method required an initial coating of mesoporous silica, followed by the loading of PS into the mesoporous layer. Another common non‐covalent physical adsorption strategy utilized the interaction between the hydrophobic PS and oleic acid (OA) layer on the UCNPs surface.^[^ ^13^, ^14^ ^]^ As the attachment of the PS molecules on the surface of UCNPs relies on weak hydrophobic interactions, the link is unstable, and PS molecules can be easily released from the surface of the UCNPs. Covalent conjugation via chemical linking can guarantee long‐term incorporation of PS molecules on the UCNPs. Still, the process requires time‐consuming and complex modification of functional groups before the chemical bonding of PS. All the methods mentioned above required extra modification or coating steps for PS loading, and none of these strategies utilize the UCNPs directly as a PS loading platform. In our study, a specific amphiphilic polymer, poly(styrene‐*co*‐maleic anhydride) (PSMA), was used to direct controllable UCNPs assembly. Adjusting the polymer‐to‐UCNPs weight ratio allows for the programmable formation of colloidally stable individual UCNPs, dimer, and trimer assemblies as well as small and big spherical UCNPs clusters. Gap zones formed when individual particles congregated and organized themselves within the UCNPs cluster can be used as channels for PS molecular loading within the clusters. With this reported assembly method, we managed to couple the UCNPs assembly process with PS loading, thereby expanding the function of controlled material assembly and simplifying the PS loading process. Furthermore, as shown in **Figure** **1**a, the fluorescence intensity of UCNPs, PS loading quantity, and tumor penetration capacity will change accordingly with each defined UCNPs cluster size, and these three factors will, in turn, influence the ^1^O_2_ production for cell killing efficacy. With programmable controlled UCNP clusters formation, we can generate UCNPs clusters of different sizes, which has the combination of all vital factors required for effective tumor mass eradication. Using this strategy, we can enhance PDT efficacy with UCNPs cluster sizes that serve as the best nanoplatforms for oncotherapy.

## 2 Results and Discussion

### 2.1 Controllable UCNPs Assembly

The basic processes of using a polymer to induce UCNPs assembly into well‐controlled clusters used in our study are illustrated in Figure 1b. The amphiphilic polymer, PSMA (Mn = 1600 g mol^−1^, the structure is shown in Figure S1, Supporting Information), was used for this assembly process.^[^ ^15^ ^]^ First, 10 mL of tetrahydrofuran (THF), 1 mL polymer in THF solution (5.25 mg mL^−1^), and 40 *μ*L UCNPs (5 mg mL^−1^, encapsulated in OA ligands in THF solution) were mixed. Following that, 1 mL of water was added using a syringe pump at the rate of 1 mL per hour. The THF within the mixture was evaporated slowly at room temperature for two days, during which UCNPs will begin to destabilize in solution. The destabilization is due to the OA ligands on the nanoparticle surface, which renders hydrophobicity to the particles causing them to aggregate to reduce interface contact with the surrounding water. Concurrently, the amphiphilic polystyrene alkane chains will spontaneously interdigitate with the alkane chains of the primary ligands located on the external surface of the assemblies through hydrophobic van der Waals interactions. The resulting spherical shaped UCNPs clusters can be easily dispersed in water. We initially chose to work with one green emission UCNPs, capped with OA on the surface synthesized as previously described^[^ ^16^ ^]^ (NaYF_4_: 20% Yb, 2% Er, diameter ≈22 nm, TEM image in Figure 1c), as an example, to demonstrate the effectiveness of this approach for fabricating UCNPs assembly. After complete THF evaporation, dynamic light scattering (DLS) was used to detect the hydrodynamic diameter changes.

We observed a large diameter peak at ≈140 nm (Figure S2, red line, Supporting Information), and it is about seven times larger than the individual UCNPs’ diameter (black line, ≈22 nm). TEM imaging revealed large UCNPs spherical clusters formation with a congregation of UCNPs particles within each cluster, and this observation matched the DLS results. The UCNPs within the big clusters continued to retain their individual character and did not fuse or integrate into larger units (Figure 1d,e). Due to their relatively large size and strong fluorescence intensity in water, we can detect and visualize spherical UCNPs clusters adequately by using fluorescence microscopy. In Figure 1f, green UCNPs clusters with narrow size distribution can be observed, and the color correlated well with the green emission of UCNPs (Figure S3, Supporting Information).

Various parameters can influence the process of UCNPs cluster formation, such as the volume ratio of THF to water, the THF evaporation rate, and the polymer to UCNPs weight ratio. However, from our experiments, the main parameter that is critical in controlling cluster size was the polymer to UCNPs weight ratio. The weight ratio used in this study varied between 4.40 and 26.25, and the ratios correspond well to cluster evolution in terms of morphologies and size of the formulated UCNPs clusters (Figure 1g). When the amount of UCNPs used for fabrication was fixed, the number of particles formed within each cluster increased with increasing polymer amount, as depicted in TEM micrographs. At polymer/UCNPs weight ratio of 4.4 (Figure 1h), the UCNPs remained individual. The DLS result of the products at this ratio is ≈24 nm, which is very close to the diameter of individual UCNPs in cyclohexane (≈22 nm). The minor increase in diameter observed may be due to the presence of the PSMA polymer sheath on the surface. The products observed were predominantly UCNPs dimers and trimers when the polymer/UCNPs weight ratio was increased to 8.75 (Figure 1i). The dimer and trimer arrangements were evident by the DLS results in Figure 1m as the peak diameter increased to ≈45 nm. UCNPs will aggregate into small and big sized spherical UCNPs clusters when the weight ratio was further increased between 13.15 and 26.25. Tetramers or UCNPs clusters containing more than four individual UCNPs each were formed when the ratio is above 13.15 (Figure 1j‐l). The corresponding hydrodynamic number distribution measured by DLS reflected the size increase from 60 to 140 nm. Statistical image analysis was performed on TEM images to quantify the percentage of individual UCNPs in dimers, trimers, and clusters formed in each sample, and at least 100 nanoparticles or more were selected for analysis (Figure 1n). At ratio 4.40, almost 100% of the samples were individual UCNPs, and at an increased ratio of 8.75, the percentages were 26%, 40%, and 34% for the individual, dimer, and trimer UCNPs, respectively. Percentages were 0.4% individual UCNPs, 4.2% dimer, 10.8% trimer, and 80.5% small UCNPs clusters (small UCNPs cluster packing number is between 4 and 10) at an increased ratio of 13.15. A further increased ratio of 17.5 yields UCNPs populations that are 1% trimer, 13.2% small clusters, and 85.8% big clusters (big UCNPs cluster packing number is above 10). Almost 100% of the individual UCNPs formed big clusters at a maximum used ratio of 26.25. We could statistically confirm that by increasing the polymer/UCNPs weight ratio from 4.40 to 26.25, the resulting clusters evolved from a significant population of the individual to mixed dimers and trimers and, lastly, to clusters of more than four UCNPs stacked per cluster. Therefore, we have used amphiphilic polymer (PSMA) to assemble UCNPs into UCNPs clusters with different sizes.

**Figure 2:**
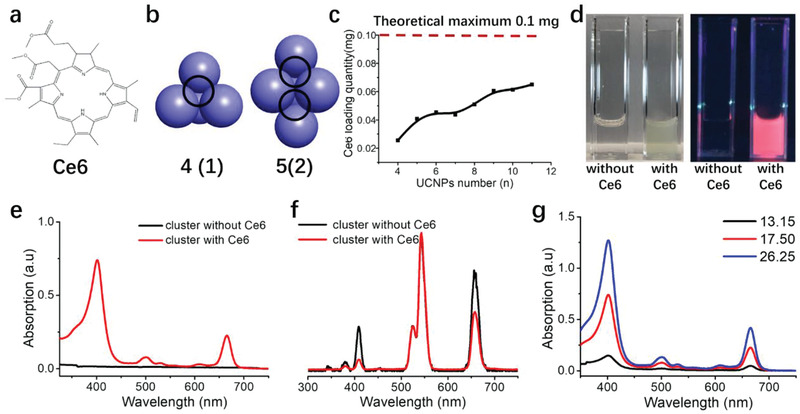
Loading Ce6 inside UCNPs clusters. a) The molecular structure of Ce6. b) The high stacking model of UCNPs clusters with different packing UCNPs number (from 4 to 5). The number in parentheses signifies the average space number surrounded by four UCNPs. c) Theoretical maximum Ce6 loading quantity within UCNPs clusters packed with different UCNPs number. d) UCNPs clusters with and without Ce6 loading. The picture on the left was taken in standard lighting, and the right photo was taken in a 365 nm UV environment. e) UV absorption of UCNPs clusters with and without Ce6 loading. f) Emission spectra of UCNPs clusters in water solution (with and without loaded Ce6) excited with 980 nm laser. g) UV absorption of different UCNPs clusters loaded with Ce6 formed at different polymer/UCNPs weight ratio. The total concentration of UCNPs clusters is 1 mg mL^−1^.

The polymer and the UCNPs exhibited good solubility in THF at the beginning of the cluster formation process. However, the addition of water and subsequent evaporation of THF act as an antisolvent that could induce the hydrophobic poly(styrene) moieties of PSMA to spontaneously interdigitate with the alkane chains of oleic acid (OA) located on the external surface of UCNPs via hydrophobic van der Waals interaction. As THF concentration decreased during evaporation, the PSMA‐PSMA interaction becomes more favorable compared to the PSMA‐OA interactions. The increased PSMA‐PSMA interactions probably provided a higher UCNPs‐UCNPs affinity and drove the slow aggregation of the UCNPs to reduce surface contact with water. An increase in the polymer ratio favors bigger cluster size formation as more UCNPs could aggregate together to minimize the contact surface area between hydrophobic polymer moieties and water. After successfully controlling the UCNPs cluster morphology and size using a single type of UCNPs with this assembly process, this method was further explored by using another kind of UCNPs with a different size and shape (rod‐like UCNPs with a length about 32 nm, Figure S4, Supporting Information).

### 2.2 Loading Ce6 inside UCNPs Clusters

We postulated that when spherical UCNPs aggregate together during assembly to form UCNPs clusters, the gap zones created between the convening UCNPs can be used as a channel for loading small molecules such as the PS, Ce6. We proceeded to use hydrophobic Ce6 (structure shown in **Figure** 2a) to test our hypothesis. Ce6 was added along with the polymer and THF solution during the process when the UCNPs assemble into spherical clusters. UCNPs clusters composed of different numbers of individual UCNPs will, in principle, have a different number of channels formed from the gap zones between tightly organized UCNPs, leading to various Ce6 loading ability.

Figure 2b illustrates the hypothesized number of channels formed for each packing model of UCNPs clusters composed of a different number of individual UCNPs. We defined UCNPs packing as a high packing density. When four individual UCNPs packed together to form a tetrahedron, there would be one channel formed in the middle of the clusters surrounded by these four UCNPs. Subsequently, when the individual UCNPs number increased to five, there would be two of such channels for Ce6 loading. The number of loading channels would increase with the increasing number of individual UCNPs packing together within one cluster. Furthermore, Ce6 molecules can be potentially loaded in all the packing models at a fixed UCNPs quantity of 1 mg (Figure 2c). The calculated theoretical maximum loading quantity is 0.1 mg Ce6 per 1 mg UCNPs. We postulated that during the THF evaporation process, both the UCNPs and hydrophobic Ce6 will not be able to disperse well in water and would aggregate together to form sediments. However, the advantage of the PSMA polymer is that it also functions as a large sheath of amphiphilic surfactants that encase and stabilize the assembled UCNPs adequately in water, preventing the formation of sediments and facilitating the formation of a well‐dispersed UCNPs cluster solution. The loaded Ce6 is contained inside the organization of the cluster via hydrophobic interactions with the OA found on the nanoparticle surface. Polymer/UCNPs weight ratio of 26.25 was henceforth chosen as a model to characterize the Ce6 loading within UCNPs clusters in the following experiments. The UCNPs clusters after Ce6 loading displayed a green color in water (Figure 2d), which matched well with the green shade of free Ce6. UCNPs clusters loaded with Ce6 exhibited a bright orange‐red coloration when exposed under UV (364 nm) irradiation as Ce6 can absorb UV light at around 400 nm and displayed an emission peak at ≈725 nm (Figure S5, Supporting Information). We then used UV absorption spectra to quantify the amount of Ce6 loaded in 1 mg of UCNPs clusters in solution. UCNPs clusters with loaded Ce6 exhibited absorption peaks at ≈400 and 675 nm, but empty UCNPs clusters devoid of Ce6 had no UV absorption signal at the same two peaks (Figure 2e). The calculated Ce6 concentration was 26.21 × 10^−6^ m at UCNPs clusters concentration of 1 mg mL^−1^, indicating that 1 mg of UCNPs clusters were loaded with 0.0167 mg of Ce6 during assembly.

Emission spectra of UCNPs clusters with Ce6 showed a slight peak intensity decrease at ≈425 and 675 nm due to the transfer of energy from UCNPs, which is absorbed by Ce6 (Figure 2f). No peak intensity difference was observed at 575 nm, as Ce6 does not characteristically display adsorption at this peak. Therefore, we can ascertain that Ce6 was successfully loaded within the UCNPs clusters and that UCNPs within the clusters are capable of resonance energy transfer to loaded Ce6. This energy transfer is vital for UCNPs cluster functionality as it could potentially be used for PDT to eradicate cancer cells. UV absorption spectra can be used to quantify the Ce6 loading ability of UCNPs clusters. As represented in Figure 2b,c, different UCNPs packing numbers will lead to various Ce6 loading capacity. When UCNPs clusters from decreasing polymer/UCNPs weight ratio (26.25 to 13.15) was measured using UV absorption spectra, the UV absorption peaks at ≈400 and 675 nm displayed concomitant reduction indicating a trend of declining Ce6 loading within the UCNPs clusters of decreasing size. This observation correlated well with our packing model. From this point, the Ce6 are mainly trapped inside the channels within UCNPs clusters. If the PS molecules are primarily positioned on the surface of UCNPs, the smaller UCNPs cluster will have a larger surface area that could load more PS molecules compared with bigger UCNPs clusters. This hypothesis is contrary to the experimental results in Figure 2g. Compared to other conventional methods of loading Ce6 to UCNPs, the stability of the assembled UCNPs clusters (Figure S6, Supporting Information) that we generated using our strategy displayed versatility and programmability for PDT applications. The loaded Ce6 can absorb the emission from UCNPs, and therefore the UCNPs clusters loaded with Ce6 can be functioned for PDT. We proceeded to compare the ability of the UCNPs‐Ce6 clusters of varying sizes (single, small and big) in delivering PS in a 3D microtumor spheroid model, singlet oxygen, ROS generation under NIR light stimulus, and tumor‐killing efficacy using bladder cancer cell lines.

### 2.3 Small Clusters Size is the Most Optimum Nanocarrier for Ce6 Transport

**Figure 3:**
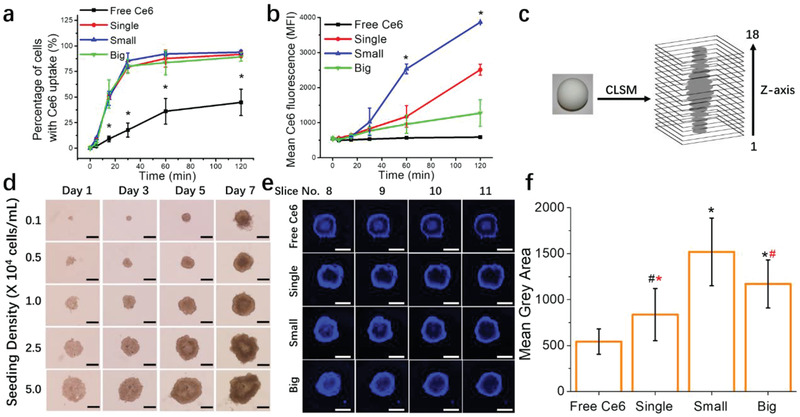
The role of UCNPs clusters sizes in intracellular uptake and penetration in 3D organoids. MB49 monolayer cells were treated with free Ce6 or clusters containing 2.5 × 10^−6^ m of Ce6 for 2 h, and Ce6 fluorescence in cells was detected via flow cytometry. a) Flow data analysis is represented as the percentage of cells with Ce6 fluorescence (* denotes significance compared to single, small and big groups), and b) mean Ce6 fluorescence intensity over time (* denotes significance compared to free Ce6, single and big groups). 3D MB49 spheroids were treated with free Ce6 or clusters containing 40 × 10^−6^ m of Ce6 for 2 h before confocal microscopy. c) Schematic of z‐stack image acquisition of spheroids. The entire depths of spheroids were captured in 18 layers of z‐planes images with 23.9 µm intervals between each plane. d) Characterization of MB49 spheroids. Phase‐contrast images of MB49 spheroid growth for seven days in culture with different seeding concentration (cells mL^−1^, 0.2 mL plated each well). Images were obtained at 4x objective. Scale bar, 500 µm. e) Slice 8 to 11 are included in the figure as they are representative of the spheroid equator. f) Ce6 fluorescence signals (mean grey area) within the equator sections (slice 8–11) of each spheroid were calculated using Image J software (black *, # denotes significance compared to free Ce6 and red *, # denotes significance compared to small group). Imaging was performed on a Zeiss LSM710 laser scanning microscope using a 5x objective lens. Scale bar, 500 µm. Data represented as mean ± SD. Experiments were performed twice in duplicates (*n* = 4). # *p* < 0.05, *p < 0.005. One‐way ANOVA with a Bonferroni post‐hoc analysis was used for comparison among multiple groups.

The programmable assembly of UCNPs for Ce6 loading in various cluster sizes serves a dual function; i) as a carrier to transport Ce6 into the tumor mass at higher magnitudes and ii) for photon upconversion of deep penetrating NIR light to visible and UV regions of the spectrum. The role of cluster size in the transport of Ce6 payload into cellular systems was investigated in vitro. Both monolayer and 3D MB49 organoids were employed to dissect the temporal and spatial kinetics of each UCNPs‐Ce6 cluster's size (single = 24 nm, small = 60 nm, big = 120 nm, *ζ*‐potential = −46 ± 2.1) in facilitating the cellular entry of loaded Ce6 and infiltration in 3D tumor masses. The use of both monolayer and 3D spheroids will provide better resolution of the PS penetration at the cellular level. We chose bladder cancer as a disease model because we have an established mouse orthotopic model.^[^ ^17^ ^]^ The orthotopic model allows for the administration of nanoparticles directly into the bladder at high concentrations, lowering the systemic exposure to the formulated nanoparticles. Furthermore, three bladder cancer cell lines were reported to form 3D spheroids for drug delivery studies.^[^ ^18^, ^19^, ^20^ ^]^ As such, most experiments were performed at 2 h treatment timepoint as this will reflect the clinical practice of leaving intravesical drugs for bladder cancer therapy to dwell for at least 2 h before bladder voidance. Flow cytometry was employed to detect the internalized Ce6 fluorescence after UCNPs cluster treatment. There are two types of data obtained from flow cytometry analysis in our study: percentage cell population that achieved intracellular Ce6 delivery and the quantity of Ce6 within cells indicated by Ce6 fluorescence, which is expressed as geometric mean. The percentage population of cells with internalized Ce6 displayed comparable uptake kinetics between the UCNPs‐Ce6 cluster groups (single, small, and big) with a rapid increase in the percentage of Ce6 positive cells from 5 (1.8–9.4%) to 30 min (79.3–85.5%). Beyond 30 min, UCNPs‐Ce6 clusters treated groups reached a saturation point, and the increase in percentages of Ce6 positive cells was marginal up to 2 h (89.3–94.0%). In contrast, the velocity of Ce6 uptake in the free Ce6 group was lethargic, and the percentages of Ce6 positive cells were significantly lower at 10 to 120 min treatment time (17.8–44.8%) compared to UCNPs‐Ce6 clusters treated groups (**Figure** **3**a).

A different pattern emerged when we looked at the geometric mean or Ce6 fluorescence intensity data from the same experiment. Despite having similar percentages with positive Ce6 levels, the mean fluorescence differs between the single, small, and big clusters. Between 10 and 120 min, mean Ce6 fluorescence was the highest for small clusters, and the order for the rate of fluorescence increase from highest to lowest is small > single > big > free Ce6 (Figure 3b). Currently, it is unknown if longer treatments would result in saturation of intracellular Ce6 fluorescent levels for all groups. However, we have performed a comparison at 14 h between free Ce6 and big UCNPs‐Ce6 clusters and observed via confocal microscopy that the big UCNPs‐Ce6 clusters treated group retained a higher level of Ce6 fluorescence compared to free Ce6 (Figures S7 and S8, Supporting Information).

Next, we examined whether the relationship between UCNPs‐Ce6 clusters sizes and Ce6 transport remained equivalent in the 3D organoid system. Using Ultra‐low attachment plates to generate 3D spheroids has many advantages. The benefits include ease of production that is not time‐consuming, low‐cost, reliable reproducibility, and the use of a biological system closely resembling a tumor.^[^ ^21^, ^22^ ^]^ We aimed to observe the penetration of Ce6 signals in MB49 spheroids via confocal microscopy and use z‐stack scanning to acquire images of the spheroids along their entire depth (Figure 3c). Spheroids generated from established cell lines using the ULA plates need to be characterized before use to confirm reproducibility between batches. The use of ULA plates for generating MB49 spheroids has never been reported. Albertó and colleagues reported the use of the liquid overlay technique to generate MB49 spheroids, but the method required agarose coating of the culture vessel and 14 days growth period.^[^ ^23^ ^]^ Figure 3d shows representative phase‐contrast images of MB49 spheroids seeded at various cell densities. Using 96‐well ULA plates for spheroid culture will generally result in the growth of one spheroid per well. Spheroids grown on ULA plates can be used from day three onward. Still, it is noteworthy that an increase in the spheroid growth period and an increase in seeding density can lead to irregular and ellipsoidal shaped spheroids (Figure 3d and Figures S9, S10, and S11, Supporting Information).

To analyze C6 penetration, we grew MB49 spheroids for three days at 2.5 × 10^4^ cells mL^−1^ density. At this stage, MB49 spheroids remained high in viability with a slight necrotic core (≈13.2%, Table S1, Supporting Information). The viability is a significant factor when studying the transport of nanocarriers in 3D spheroids as necrotic cells will be devoid of active transport processes, and only free drugs that enter by passive transport will be able to permeate the necrotic region. For this experiment, 40 × 10^−6^ m of equivalent Ce6 concentration was used for all the groups, and z‐stack slices from 8–11 were chosen to represent the central equatorial core of the spheroids (Figure 3e). Concentrations of UCNPs‐Ce6 particles used for the equivalent of 40 × 10^−6^ m Ce6 are 1.88, 1.39, and 1.16 mg mL^−1^, respectively, for single, small, and big clusters. Treatment with 40 × 10^−6^ m Ce6 and corresponding high UCNPs clusters concentration for 4 h induced dark toxicity at 24 h assay (Figure S12e, Supporting Information). However, we proceeded with these concentrations for confocal microscopy experiments because intracellular signals from low concentrations of free Ce6 were very difficult to detect. At 40 × 10^−6^ m concentration, free Ce6 treatment can be visibly detected at 2–4 h, which allowed for correlation of confocal microscopy observations with PDT efficacy performed at 2 h. The spheroids chosen for penetration assay were consistent with similar diameter, roundness, and solidity to ensure fair comparisons of Ce6 infiltration. UCNPs‐Ce6 cluster particles all showed superior Ce6 permeation within the spheroid compared to free Ce6. The intensity of Ce6 penetration within the spheroids between UCNPs‐Ce6 groups interestingly does not adhere to the same pattern observed in conventional monolayer cells. Figure 3f demonstrates the graphical quantification of Ce6 fluorescence signal intensity within the central equatorial core of each respective sample. The Ce6 fluorescent intensity levels are in the order of small > big > single > free Ce6.

It is well reported that 3D spheroid arrangement supports the formation of extracellular matrix (ECM) layer present in biological tissues but are absent in established monolayer cell lines. For bladder 3D organoids, increased expression of ECM components such as E‐cadherin, CK‐20, and the tight junction protein zonula occludens‐1 was observed in the 3D cellular conformation when compared to 2D culture.^[^ ^24^ ^]^ ECM components are potential penetrative barriers for nanoparticle infiltration in tumor mass.^[^ ^25^ ^]^ Such ECM layers are typically hydrophilic and may have obstructed the penetration of hydrophobic Ce6 into the spheroid core, which depends on diffusion‐driven penetration. In contrast, UCNPs‐Ce6 clusters are not able to permeate through the cell membrane despite their small size due to their polarity. UCNPs typically employ endocytic pathways for cellular entry, and UCNPs‐Ce6 clusters may similarly access cells via bulk entrance endocytic channels allowing for the penetration of the UCNPs‐clusters formulations on the spheroid surface.^[^ ^26^, ^27^ ^]^ In contrast to its performance in monolayer cultures, big UCNPs‐Ce6 clusters displayed improved transport of Ce6 in 3D MB49 spheroids when compared to the single cluster group. This observation suggests that while the size of the UCNPs‐Ce6 cluster may be a limiting factor in monolayer cells, penetration within 3D spheroids may also rely on other entry mechanisms such as diffusion. Big clusters displayed enhanced carrier functionality in 3D environment probably because they carry a larger Ce6 payload compared to single clusters,

There may also be other underlying UCNPs' physicochemical properties besides their size and zeta potential that aids in the penetration of UCNPs clusters in the depths of 3D spheroids. For example, it is known that nanoparticles can adsorb protein corona on their surfaces, which may have an effect on their penetration and uptake in tumor masses.^[^ ^28^ ^]^ Since this was not investigated in our current study, it is unclear if the transfer of UCNPs‐Ce6 clusters to culture media that contains serum proteins may have resulted in protein corona formation on the clusters surfaces. It is also important to note that we detect Ce6 penetration within the spheroids and not the UCNPs directly. For small and big clusters, their packing structure, which allows for larger amounts of Ce6 loading, could have resulted in increased Ce6 delivery into the spheroid core without a concomitant increase in UCNPs clusters particle penetration. Between the small and big clusters, their sizes make a difference in carrier capability. The small clusters exhibited better Ce6 delivery into the spheroids’ equatorial regions compared to big clusters. It is well known that nanoparticles that are smaller than 100 nm in hydrodynamic diameter exhibited enhanced permeability and retention effect. Hence, they can preferentially infiltrate into tumor masses compared to a similar nanoparticle type of larger size.^[^ ^29^ ^]^ Nevertheless, our work indicates that we can create larger particles that do not compromise on nanocarrier efficacy using our controlled UCNPS assembly method.

### 2.4 Big Clusters are the Most Potent Inducers of Singlet Oxygen Release

**Figure 4:**
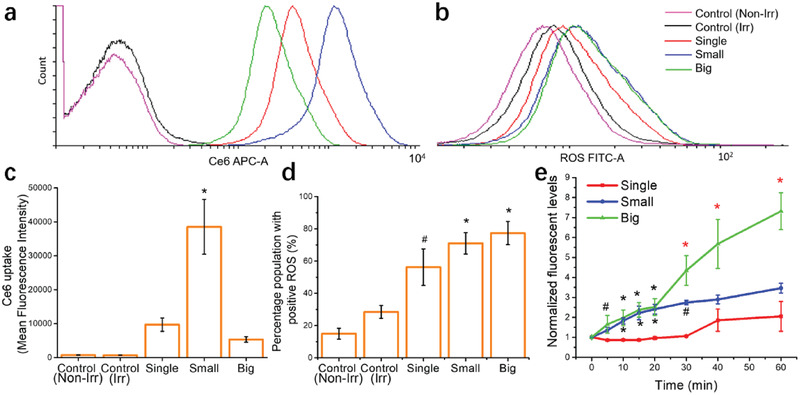
Photoactivation of different clusters sizes and their effects on ROS induction. MB49 monolayer cells were treated with 0.5 mg mL^−1^ UCNPs‐Ce6 clusters for 2 h, followed by 30 min irradiation with 980 nm continuous wave laser (2.5 W cm^−2^). Ce6 uptake and ROS levels were detected with CM‐H_2_DCFDA via flow cytometry. Histogram showing a) Ce6 uptake and b) ROS production in MB49 cells treated with different UCNPs‐Ce6 clusters sizes. c) Mean Ce6 fluorescence intensity (* denotes significance compared to all groups) and d) percentage of cell population with positive ROS levels (#, * denotes significance compared to control, irradiated group). e) Clusters were irradiated for 60 min with 980 nm continuous wave laser to study the temporal control of singlet oxygen generation. Black #, * denotes significance compared to single group and red #, * denotes significance compared to single and small groups) Cluster concentration used was 0.5 mg mL^−1^, and Ce6 concentrations were 10, 12, and 17 × 10^−6^ m for single, small and big clusters respectively. Data were represented as mean ± SD. Experiments were performed twice in duplicates (*n* = 4). # *p* < 0.05, *p < 0.005. One‐way ANOVA with a Bonferroni post‐hoc analysis was used for comparison among multiple groups.

The hallmark of PDT reaction is the formation of highly reactive oxygen species that can directly induce cellular toxicity. Photoactivation of Ce6 involves a Type II PDT reaction whereby energy transfer from Ce6 to molecular oxygen results in the formation of highly reactive ^1^O_2_.^[^ ^30^ ^]^ Superoxide anion can be formed from ^1^O_2_, leading to the cascade formation of other ROS, such as hydrogen peroxide, hydroxyl, and hydroperoxyl radical.^[^ ^31^ ^]^ Thus, it was mandatory to investigate and compare the ROS and ^1^O_2_ generation of each cluster size in response to NIR light irradiation. MB49 cells were treated with 0.5 mg mL^−1^ UCNPs‐Ce6 clusters for 2 h, followed by irradiation with 980 nm continuous wave laser for 30 min before ROS detection via flow cytometry. At this concentration, 100% of the sample population displayed Ce6 positive signals (**Figure** **4**a). Small clusters repeatedly proved to be the most potent vehicle for Ce6 transport in MB49 monolayer cells, and the trend in order of decreasing Ce6 intracellular fluorescence levels remained the same for MB49 monolayer cells: small > single > big (Figure 4a). ROS histogram overlay showed overlapping histograms for small and big clusters (Figure 4b, blue and green line) with a lower single clusters histogram shift.

Despite the significant contrast of mean Ce6 fluorescence between small and big clusters (Figure 4c, 38 539 vs 5329 MFI), the percentage of ROS positive cells are similar (Figure 4d, 71 vs 77.4%). We proceeded to compare the Ce6 activation efficiency of each cluster geometry in the presence of the NIR irradiation source by measuring ^1^O_2_ release. Small and big UCNPs‐Ce6 clusters appear to function similarly up to 20 min, but ^1^O_2_ levels increased significantly for the big clusters group from 30 to 60 min irradiation time. Single clusters displayed an increase in ^1^O_2_ levels only at 40 and 60 min, but the levels were relatively low compared to small clusters and significantly lower than big clusters ^1^O_2_ generation capacity (Figure 4e).

Given that ^1^O_2_ has a short half‐life,^[^ ^32^ ^]^ those formed in the central core of the big cluster cannot diffuse out in time. It is possible that only when the irradiation is long enough (above 20 min, Figure 4e), the potency of the big clusters can be observed. The superior upconversion property of the big cluster was expected due to its immense structure, and it could emit higher fluorescence energy when activated by the NIR light source. Furthermore, big clusters have the potential to carry higher Ce6 payload within its channels. Thus, prolonged NIR irradiation could help to activate encapsulated Ce6 to produce and accumulate ^1^O_2_, resulting in increased ^1^O_2_ diffusion into the surrounding solution. Additionally, the surface area to volume ratio (SA/Vol) of the clusters could have influenced the upconversion properties of UCNPs. Big UCNPs clusters have a lower SA/Vol ratio compared to small UCNPs clusters (0.05 vs 0.1 nm^−1^, respectively), and a lower SA/Vol ratio could ensure the elimination of water‐mediated luminescence quenching of the UCNPs on the surface of UCNPs cluster.^[^ ^33^ ^]^ Furthermore, Shan and colleagues demonstrated that particles of lower SA/Vol ratio displayed higher upconversion luminescence compared to particles of the same shape but with a higher SA/vol ratio.^[^ ^34^ ^]^ It is plausible that the increased ^1^O_2_ formed in the big cluster samples from 20 to 60 min coincided with increased upconversion luminescence intensity over time. However, we do not have the data to support this hypothesis.

The results from the ^1^O_2_ assay could explain the similarities in ROS inducing profiles for small and big cluster groups despite the differences in internalized Ce6 fluorescence. ROS from both Type I and Type II PDT reactions can cause lipid peroxidation (LPO), an event by which the polyunsaturated fatty acids (PUFA) found in cell membranes undergo oxidative damage in the presence of free radicals.^[^ ^35^ ^]^ Singlet oxygen has been shown to participate in the propagation phase of cellular LPO events and the formation of downstream intermediary LPO products such as peroxyl radicals and peroxynitrite anions.^[^ ^36^, ^37^ ^]^ Both these products can be detected with the carboxy‐H_2_DCFDA reagent used for ROS analysis. Hence, we hypothesize that the potent nanocarrier ability of the small UCNPs clusters is nullified by the significantly superior upconverting properties of the big clusters resulting in similar ROS levels when irradiated with NIR light.

The subcellular distribution of PS is an essential determinant of their efficacy, more so than their ^1^O_2_ quantum yield.^[^ ^38^ ^]^ Characteristically, ^1^O_2_ is very short‐lived with a lifetime of 40 ns and a diffusion radius of 20 nm.^[^ ^32^ ^]^ Hence, their site of generation is critical as the time interval at which they can exert their damaging effects is very limited. Localization of Ce6 to the mitochondria is most advantageous for PDT as the organelle contains high concentrations of oxygen due to their role in oxidative phosphorylation and are also critical regulators of apoptotic cell death.^[^ ^32^ ^]^ Singlet oxygen‐induced damage to the mitochondria can trigger an immediate apoptotic response. Therefore, we studied the subcellular localization of big UCNPs‐Ce6 clusters to the mitochondria. Colocalization analysis was done with Image J software to compute the Pearson correlation coefficient values. Values that are closer to 1 indicate a higher probability that analyzed pixel signals between 2 groups were closely located. The Pearson correlation coefficient showed increased Ce6 localization to the mitochondria for big UCNPs‐Ce6 clusters treated samples in MB49 and RT4 (with a minimum factor of twofold) but not for T24 and UMUC3 cell lines (Figure S8, Supporting Information). The weak signal visibility of free Ce6 detected in confocal microscopy is a limitation in this experiment. To improve the Ce6 signal visualization, a higher concentration of free Ce6 and longer incubation time is necessary. However, we chose to adhere to lower incubation time to reflect clinical practice. Hence, the increase in Pearson's correlation observed in MB49 and RT4 cell line may simply be attributed to a rise in Ce6 particles internalized in monolayer cells and not due to mitochondrial specificity of UCNPs encapsulated Ce6. Nevertheless, encapsulation increased the likelihood of mitochondrial proximity in MB49 and RT4 cell lines, and this is useful for PDT applications in oncotherapy. Low levels of ^1^O_2_ produced in the mitochondria can provide sufficient toxicity by inducing a sharp decrease in mitochondrial membrane potential, triggering apoptosis for tumor eradication.^[^ ^39^, ^40^ ^]^

### 2.5 Small UCNPs‐Ce6 Clusters are Most Efficient at In Vitro Toxicity

**Figure 5:**
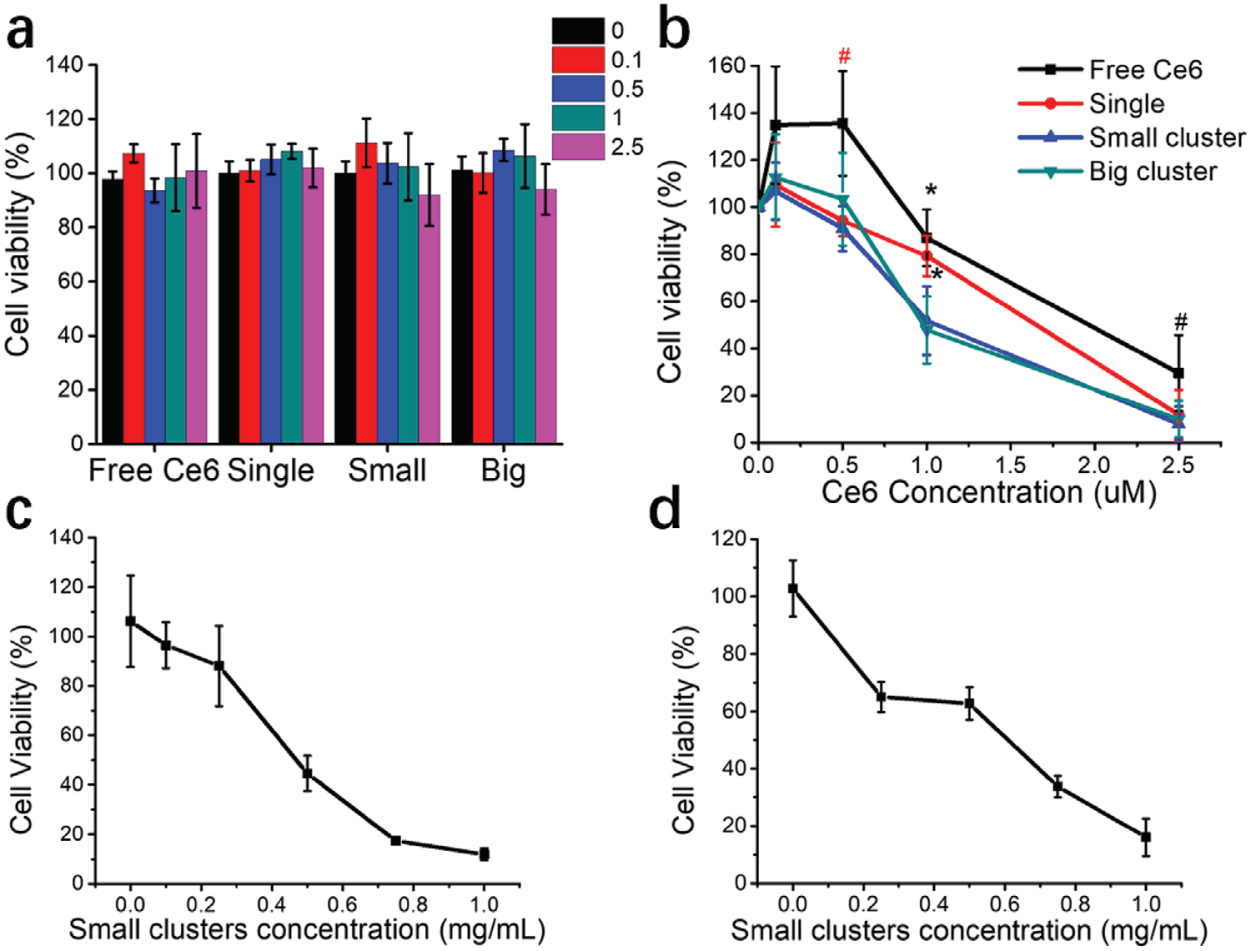
In vitro PDT efficacy of UCNPs‐Ce6 clusters. a) Dark toxicity assay was performed for 2 h on MB49 monolayer. See Table S2, Supporting Information for respective cluster concentrations. b) In vitro visible light PDT efficacy of UCNPs‐Ce6 clusters formulations in MB49 monolayer cell line. Red # denotes significance compared to single, small, and big groups, and black #, * denotes significance compared to small and big group. Treatment with UCNPs‐Ce6 formulations for 2 h was done, followed by irradiation with a dual‐LED device (405 and 660 nm) for 30 min. c) In vitro NIR PDT efficacy with small UCNPs‐Ce6 clusters on MB49 cell line. d) In vitro NIR PDT efficacy with small UCNPs‐Ce6 clusters on MB49 spheroids. MB49 monolayer cells or 3D spheroids were treated with small UCNPs‐Ce6 clusters at various concentrations for 2 h, followed by irradiation with 980 nm continuous wave laser (2.5 W cm^−2^) for 30 min. Data were represented as mean ± SD. The experiment was performed thrice in duplicates (*n* = 6) for visible light PDT and twice in duplicates (*n* = 4) for NIR PDT. #*p* < 0.05, *p < 0.005. One‐way ANOVA with a Bonferroni post‐hoc analysis was used for comparison among multiple groups.

Next, we investigated the in vitro PDT efficacy in the presence of a visible light source. This experiment aims to ascertain and compare the UCNPs‐Ce6 clusters’ function as a nanocarrier for Ce6 delivery as Ce6 is directly activated by visible light. Dark toxicity of the big UCNPs‐Ce6 clusters was previously performed for 14 h on UMUC3 and MB49 monolayer cells at concentrations ranging from 0.1‐1 mg mL^−1^ and no toxicity were observed (Figure S12a,b, Supporting Information). Samples were treated with equivalent concentrations of Ce6 ranging from 0.1–2.5 × 10^−6^ m to ensure equal photosensitizer exposure despite different concentrations of UCNPs‐Ce6 particles (Table S2, Supporting Information). A wired LED device connected to a DC power supply was used as an irradiation source. The safety and efficacy of a wireless, remote‐controlled version of this device in treating subcutaneously implanted bladder tumors in mice had already been demonstrated.^[^ ^41^ ^]^ The radiant power is kept at 1 mW cm^−2^ throughout this study at 30 min irradiation period. At the abovementioned Ce6 concentrations, no dark toxicity was observed for MB49 monolayer PDT (**Figure** **5**a), but there was significant dark toxicity when MB49 spheroids were treated with 5 × 10^−6^ m small UCNPs‐Ce6 clusters (79.6%, Figure S13a, Supporting Information). Visible light source irradiation with corresponding empty big UCNPs clusters concentrations did not display any toxicity indicating that any PDT toxicity observed was induced by encapsulated Ce6 (Figure S12c,d, Supporting Information). For all treatment groups, PDT toxicity occurred in a dose‐dependent manner (Figure 5b,c). Small and big UCNPs‐Ce6 clusters performed similarly in MB49 monolayer cells despite their significant disparity in Ce6 delivery (Figure 3b).

The single UCNPs group displayed a slight lag at 1 × 10^−6^ m concentration, but their performance at 2.5 × 10^−6^ m was similar compared to small and big clusters groups. This result correlated with cellular uptake studies (Figure 3a) as the percentage population of cells with Ce6 uptake at this concentration for 2 h are similar for all cluster groups and have reached the level of saturation (91.8%, 94.0%, and 89.3% for single, small, and big clusters respectively). When ROS levels reach an overwhelming threshold such that cellular antioxidative mechanisms could not keep up with their ROS scavenging activities, the resulting oxidative stress could lead to the initiation of cellular death process.^[^ ^42^ ^]^ Hence, it is plausible that despite differences in intracellular Ce6 between all samples, irradiation with visible light at 30 min resulted in an increase in ROS levels beyond the abovementioned threshold for cellular death. We proceeded to perform the same PDT assay with MB49 spheroids.

Currently, there is no consensus on the ideal spheroid diameter for studying cytotoxic effects. Several research groups chose to work with spheroids with a medium diameter ranging between 300–500 µm. Smaller spheroids (<200 × 10^−6^ m) do not develop chemical or proliferative gradients.^[^ ^43^, ^44^ ^]^ In comparison, larger spheroids of more than 500 µm in diameter may develop a secondary necrotic core, which will decrease the accuracy of cytotoxicity assay.^[^ ^45^ ^]^ The size range of 300–500 µm also fits the requirements for gradients of oxygen, nutrients, and proliferation rate that are essential to ensure biological relevance with in vivo conditions. For both MB49 and RT4 cells, 0.5 × 10^4^ cells mL^−1^ seeding density can be used to generate spheroids between 300–500 µm for toxicity assay at day three post‐seeding (Table S3, Supporting Information). PDT toxicity for all groups on MB49 spheroids occurred in a dose‐dependent manner (Figure S13b, Supporting Information), and the results correlated with the spheroid penetration assay (Figure 3e,f). Comparing the PDT results between monolayer cells and spheroids, two observations are noteworthy. First, the cellular arrangement in 3D spheroids resulted in PDT resistance with free Ce6 treatment (Tables S4 and S5, Figure S14c,d, Supporting Information). This observation correlated well with 3D spheroid systems developed from other cell types.^[^ ^46^, ^47^ ^]^ However, the treatment of MB49 and RT4 spheroids with UCNPs‐Ce6 clusters was able to overcome this resistance as observed by the consistent IC50 values between the monolayer and 3D spheroids experiments for clusters treated groups (Tables S4, S5, Figure S14c,d, Supporting Information). The PDT resistance seen with free Ce6 could be caused by a combination of insufficient Ce6 penetration and the presence of marked hypoxic core in 3D spheroids (Figure S15, Supporting Information). The ability to provide toxicity in a hypoxic environment is an essential factor as resistance to PDT could result in subsequent tumor growth. PDT resistance was observed before in studies with limited PDT efficacy being able to induce pro‐survival mechanisms and immune escape in vivo.^[^ ^48^, ^49^ ^]^

Second, treatment with small UCNPs‐Ce6 clusters leads to enhanced killing in 3D cellular conformation compared to monolayer cells. While the killing efficacy of small clusters in monolayer cells could be saturated due to its 2D environment, the smaller particle diameter could work to greater advantage in 3D systems. The ^1^O_2_ generated by Ce6 packed at the inner core of the cluster circumference must diffuse through a smaller radius to encounter and exert damage to the nearest cellular components or organelles. As cells are densely packed in 3D formation, especially within the inner layer, each cellular component in 3D spheroids is exposed to more neighboring cells than in monolayer culture.^[^ ^50^ ^]^ Hence, ^1^O_2_ formed in 3D spheroids can exert its effects on cells multiple layers deep. Small clusters also have the potential to use generated ^1^O_2_ optimally due to its larger SA/Vol ratio. There are more Ce6 molecules on the small cluster surfaces than the big cluster, and the smaller radius allows for augmented ^1^O_2_ diffusion out of the cluster sphere.^[^ ^51^ ^]^ As small UCNPs‐Ce6 clusters were found to be the best performing nanocarrier with visible light PDT, we proceeded to test its efficacy in the presence of NIR irradiation on both MB49 monolayer cells and 3D spheroids. The experiment was performed with small UCNPs‐Ce6 clusters concentration range of 0.1–1 mg mL^−1^ (Table S6, Supporting Information) for 2 h, followed by PDT with 980 nm continuous wave laser at 2.5 W cm^−2^ for 30 min. PDT toxicity was observed in a dose‐dependent manner in both monolayer MB49 cells and 3D spheroids with IC50 of 0.49 and 0.62 mg mL^−1^, respectively (Figure 5c,d). However, dark toxicity was observed with small UCNPs‐Ce6 cluster treatment in both MB49 monolayer and 3D spheroids (Figure S12f, Supporting Information). Nevertheless, a significant increase in PDT induced toxicity was observed at higher concentrations for monolayer cells (0.5 and 0.75 mg mL^−1^, Table S7, Supporting Information) and 3D spheroids (0.75 and 1 mg mL^−1^, Table S8, Supporting Information) when compared to dark toxicity data.

Subsequent efforts to investigate the effects of direct small UCNPs‐Ce6 administration in an in vivo subcutaneous tumor model in mice was undertaken to confirm if dark toxicity will be similarly observed in a living system. As displayed in Figure S12g (Supporting Information), the subcutaneous tumor treated with small UCNPs‐Ce6 clusters (2 mg mL^−1^, ≈0.1 mL) grew in volume over time and is comparable to the control saline‐treated tumor. Data of mice weight recorded during the experimental period and harvested organ masses indicated no significant systemic effects of intratumoral administration of small UCNPs‐Ce6 clusters (Figure S12h,i, Supporting Information). The dark toxicity observed in vitro can be explained by the nanocarrier efficacy of the small clusters. As noted in Figures 3b and 4c, the small clusters are excellent nanocarriers for the delivery of the Ce6 payload. The use of small clusters as Ce6 delivery platform resulted in increased levels of intracellular Ce6 that is significant in proportion when compared to big UCNPs‐Ce6 clusters, which did not exhibit in vitro dark toxicity at concentrations up to 1 mg mL^−1^ after 14 h of treatment (Figure S12a,b, Supporting Information). Ce6 can undergo spontaneous activation in solution to produce ^1^O_2_, which may have caused dark toxicity in small UCNPs‐Ce6 clusters treated cells and spheroids. However, contrary to in vitro data, no dark toxicity was observed in the in vivo subcutaneous tumor treated with small UCNPs‐Ce6 clusters at double the concentration used in vitro. The contradictory results found in this study highlights the fact that in vitro toxicity concentrations are generally not applicable for treatment in fully functioning biological systems. Two factors could explain the conflicting observations. Firstly, direct intratumoral administration into the significantly larger sized solid tumor allowed for an unsaturated and uniform distribution of the small cluster formulation within the tumor mass. Hence, intracellular Ce6 levels in vivo may not attain the level of proportions found in vitro. Secondly, ^1^O_2_ quenchers such as ascorbic acid, tryptophan, and histidine can be found circulating in the plasma fluids in the interstitium, and extracellular fluids in living systems, thereby preventing the cytotoxic effects of spontaneous ^1^O_2_ formation from small UCNPs‐Ce6 clusters treatment.^[^ ^52^, ^53^ ^]^ Nevertheless, we have demonstrated with our experimental results that using our method of controlled assembly of UCNPs, the size of the cluster structure can be programmed for optimal nanocarrier functionality and PDT efficacy with both visible and NIR light source.

## 3 Conclusion

In summary, we achieved controlled formation of UCNPs clusters of various sizes (individual; dimer and trimer; small and big clusters) via regulation of PSMA polymer to UCNPs weight ratio used during the assembly process. Successful loading of Ce6 within the UCNPs clusters formulations functioned for PDT application can be achieved by exploiting the gap zones formed between congregating UCNPs as channels for Ce6 packing and encapsulation. The assembly strategy used is simple, reliable, and is versatile in terms of generating clusters of varying geometries for potential PDT applications.

In the present study, we showed that small‐sized UCNPs‐Ce6 clusters with an approximate diameter of 60 nm were the best performing nanocarrier when compared to single (≈24 nm) and big (≈120 nm) UCNPs‐Ce6 clusters. The result is interesting as diffusion of nanocarriers in tumor masses is thought to be inverse to the molecular weight, which meant that larger nanoparticles would diffuse slowly than low molecular weight drugs. However, this does not apply to these UCNPs‐Ce6 formulations suggesting that the penetrative barrier preventing free Ce6 diffusion into spheroids core is overcome by UCNPs encapsulation using our assembly method. The choice of clusters size for in vivo use needs careful consideration. The selected particle size must exhibit the right balance of penetrative capacity in 3D microtumor mass, ^1^O_2_, and ROS formation under NIR light and cellular toxicity. Small and big UCNPs‐Ce6 particle formulations are the ideal choice due to their geometries and their packing structure that can accommodate more substantial amounts of Ce6 within their gap channels. In so far, they have exhibited different strengths as a material for cancer PDT application. While the small UCNPs‐Ce6 clusters can deliver a more considerable amount of Ce6 into 3D MB49 microtumors, big clusters exhibited superior ^1^O_2_ generating capacity under NIR irradiation, suggesting a structure that can provide enhanced resonance energy transfer to encapsulated Ce6. However, despite the differences in their respective potency, they achieved almost equivalent cellular killing efficacy with the visible light source in MB49 3D organoids. ROS generation under NIR light stimulus was also similar for both small and big UCNPs‐Ce6 clusters. The NIR induced in vitro killing efficacies between small and big clusters, which is not investigated in our present work, must be compared before proceeding to in vivo studies.

Even though 3D spheroids are improved cellular models for biological tissues, they lack the heterogeneity found in the tumor microenvironment.^[^ ^54^ ^]^ Furthermore, spheroid models are devoid of a vascular system. A well‐vascularized tumor microenvironment could increase the penetration of investigated nanocarriers. PDT efficacy could also improve due to the supply of oxygen by the vascular system for prolonged PDT reaction. As such, PDT strategies using UCNPs‐Ce6 clusters formulations for effective eradication of tumors need to be adaptive for maximum success. The advantage of our described method of UCNPs assembly is the versatility of clusters products that can be generated for PDT use. The use of different sized clusters with all crucial factors such as Ce6 loading and penetration capacity can be studied to utilize their respective strengths fully. Additionally, the development of an optimum treatment schedule as a strategy to ensure complete disease remission must also be considered for the clinical treatment regimen. A combination light source approach (UV/vis + NIR) is advantageous as a significantly lesser concentration of UCNPs‐Ce6 clusters needs to accumulate in tumor mass to induce toxicity with the visible light source. The accompanying NIR light source will then target greater tumor depths, increasing the PDT target area. Implantable LED devices for treating deep‐seated and larger tumors have been reported. Such devices can be employed to improve PDT strategy. Our results showed that the novel UCNPs‐Ce6 cluster formulations show promise for use as nanotherapeutics in cancer PDT, and the assembly method can pave the way for UCNPs based applications in other fields.

## Conflict of Interest

The authors declare no conflict of interest.

## Supporting information

Supporting Information
