# Supplementary material for: Controllable Assembly of Upconversion Nanoparticles Enhanced Tumor Cell Penetration and Killing Efficiency
Source: Adv Sci (Weinh). 2020 Nov 7;7(24):2001831. doi: 10.1002/advs.202001831 (PMC7739948; doi:10.1002/advs.202001831)
Supplement: Supplementary file 1 — Supporting Information [file ADVS-7-2001831-s001.pdf]

WILEY-VCH

© 2020 Wiley-VCH GmbH

ADVANCED SCIENCE
Open Access

Supporting Information

for *Adv. Sci.*, DOI: 10.1002/advs.202001831

Controllable Assembly of Upconversion Nanoparticles Enhanced Tumor Cell Penetration and Killing Efficiency

*Zhen Zhang, Juwita Norasmara Rahmat, Ratha Mahendran and Yong Zhang**

© 2020 Wiley-VCH GmbH

Supporting Information

Controllable assembly of upconversion nanoparticles enhanced tumor cell penetration and killing efficiency

Zhen Zhang, Juwita Norasmara Rahmat, Ratha Mahendran and Yong Zhang[*]

Supporting Information

Controllable Assembly of Upconversion Nanoparticles Enhanced Tumor Cell Penetration and Killing Efficiency

Zhen Zhang, Juwita Norasmara Rahmat, Ratha Mahendran and Yong Zhang[*]

Dr. Z. Zhang, Dr. J.N. Rahmat, and Prof. Y. Zhang
Department of Biomedical Engineering, Faculty of Engineering, National University of Singapore, Singapore 117583
Dr. R. Mahendran
Department of Surgery, Yong Loo Lin School of Medicine, National University of Singapore, Singapore 119228
Prof. Y. Zhang
NUS Graduate School for Integrative Sciences and Engineering, National University of Singapore, Singapore 117456

Z. Zhang and J.N. Rahmat contributed equally
E-mail: biezy@nus.edu.sg

Experimental Section

Materials: YCl_3•$6H_2O$ (99.9%), $YbCl_3$•$6H_2O$ (99.9%), $ErCl_3$•$6H_2O$ (99.9%), 1-octadecene (90%), oleic acid (OA) (90%), poly(styrene-comaleic anhydride) (PSMA, Mn = 1600 g mol^{-1}), chlorin-e6-trimethyl ester (Ce6) and tetrahydrofuran (THF) were purchased from Sigma-Aldrich (St. Louis, MO, USA) and used as received without further purification.

Synthesis of β-phase $NaREF_4$ (RE=Y, Yb, Er) core nanocrystals: $NaREF_4$ nanocrystals were synthesized using a standard solvent thermal synthesis method. Briefly, water was fully evaporated from a $RECl_3$ (1 mmol) aqueous solution in the stoichiometric ratio followed by the addition of 1-octadecene (15 mL) and oleic acid (6 mL). The mixture was heated at 156 ^{0} C for 10 minutes to form RE-oleate complexes. The resulting solution was then cooled to room temperature and mixed with a methanol solution (5 mL) containing NH_4F (4 mmol) and NaOH (2.5 mmol). The temperature was then raised to 120 ^{0} C for 10 minutes for complete methanol removal and degassed for 15 minutes to eliminate residual methanol and oxygen. Subsequently, the temperature of the resulting solution was raised to 300 ^{0} C for 1 hour in an atmosphere of argon, and the products were precipitated down with acetone followed by centrifugation at 8000 rpm for 10 minutes. The precipitate was washed with acetone and dispersed in cyclohexane (10 mL) for further use.

Synthesis of UCNPs clusters: UCNPs in cyclohexane solution (50 µL, 5 mg mL^{-1}) was added to THF solution (10 mL) comprising various PSMA polymer concentrations to achieve PSMA/UCNPs weight ratios of 4.4 to 26.25. The mixture was stirred vigorously for 10 minutes, followed by the controlled addition of H_2O (1 mL) via a syringe pump at the rate of 1 mL per hour. The low boiling-point THF was evaporated by continuous stirring (200 rpm) at room temperature for two days. UCNPs clusters were obtained after this process and dispersed in water. Ce6 loading process was performed similarly with Ce6 (0.05 mg) added at the start in the THF solution along with the UCNPs and polymer. The resulting solution of UCNPs-Ce6 clusters exhibits a uniform dark green color. The addition of chloroform at the last step in clusters formation served to remove excess polymer.

Cell culture: Human transitional cell carcinoma cell lines T24, RT4 (ATCC, Manassas, VA, USA) and murine urothelial carcinoma, MB49 (a kind gift from Timothy Raitliff, Purdue University) were routinely cultured in RPMI supplemented with 10% heat-inactivated fetal bovine serum (Hyclone, Logan, UT, USA) 2 mM L-glutamine (Thermo Fisher, Waltham, MA, USA) 50 U mL^{-1} Penicillin G (Thermo Fisher) and 50 µg mL^{-1} streptomycin (Thermo Fisher). UMUC3 cells were maintained in MEM media with the same supplementation as RPMI. Cells were grown at 37 °C in a 5% CO_2 atmosphere and routinely passaged when 85-90% confluent.

Spheroid formation and characterization: MB49 and RT4 cells were plated in Ultra-low attachment (ULA) 96-well round-bottom plates (Corning, NY, USA) at various seeding densities in 0.2 mL of supplemented RPMI media. The cells were centrifuged lightly at 100 x g for 3 minutes after seeding to induce cellular proximity, minimize cell death, and encourage the formation of a single spheroid. New media (50 µL) were added every three days, and spheroids were cultured for seven days. Images of all spheroids were taken daily using an Olympus IX71 microscope with a 4x objective lens attached to an Olympus DP71 camera (Tokyo, Japan). Daily spheroid images were analyzed using Image J software using a macro written by Ivanov et al. to calculate the area, maximum and minimum Ferret diameter of the spheroid as well as other spheroid parameters such as circularity, solidity, and roundness. The measured area (A) of the 2D projection of the spheroids was used to calculate the radius ($R = \sqrt{\frac{A}{\pi}}$) and the volume ($V = \frac{4}{3} R^3$) of an equivalent sphere in Microsoft Excel. Spheroid characteristics data were plotted in GraphPad Prism v6 (San Diego, CA, USA).

Spheroid Growth and Viability: Spheroids generated from 3 wells were pooled together in a microfuge tube. Centrifugation was performed at 450 x g for 10 minutes at 4 °C and the pelleted spheroids were washed once with 1x phosphate-buffered saline (PBS, 0.5

mL). Spheroids were pelleted by centrifugation followed by treatment in a 0.25% trypsin solution (0.1 mL) at 37°C for 6 minutes to dissociate the spheroids into single cells. Complete media (0.5 mL) were added to inactivate the trypsin. Cells were pelleted by centrifugation and resuspended in 20 µL of media. The cells were stained with 0.4% (v/v) trypan blue, and the numbers of total and viable cells were recorded using the TC10™ Automatic cell counter (Bio-Rad, Hercules, CA, USA). The data were analyzed and expressed as the average number of cells per spheroid and the percentage of viable cells per spheroid.

Cellular uptake of Chlorin e6: MB49 monolayer cells (5 x 10^5) were plated in 24 well plates overnight before treatment with free Chlorin e6 (trisodium Ce6, Frontier Scientific, Logan, UT, USA), single, small, or big UCNPs-Ce6 clusters. All groups contained an equal concentration of Ce6 (2.5 µM) and were treated for 5, 10, 15, 30, 60, and 120 minutes. The cells were harvested at the end of the incubation by washing twice with 1 x PBS (0.5 mL), trypsinized, and fixed in 4% formaldehyde solution (1 x PBS containing 0.5% BSA, 0.3 mL) for 15 minutes before Ce6 detection via flow cytometry (FACS Canto, BD, Franklin Lakes, New Jersey, USA) using the FACSDiva software. Data was acquired from at least 1 x 10^4 cells per sample for analysis.

Penetration of Ce6 in 3D spheroids: MB49 cells (2.5 x 10^5 cells mL^{-1}) were plated in ULA plates for three days in 0.2 mL volume to generate spheroids between 600-800 µm in diameter. Spheroids were treated with free Ce6, single UCNPs-Ce6, small or big UCNPs-Ce6 clusters containing 40 µM Ce6 for 2 hours before confocal microscopy. Z-stack imaging of the entire depth of each spheroid with Zeiss LSM710 laser scanning microscope (Carl Zeiss AG, Oberkochen, Germany) was performed using a 5x objective lens. The images were analyzed for mean Ce6 fluorescence within the spheroid boundary using Image J software.

ROS analysis: MB49 monolayer cells (1 x 10^5) were plated in 48 well plates overnight (Nunc, Rochester, NY, USA) before treatment with 0.5 mg mL^{-1} UCNPs-Ce6 particles (single, small and big clusters) for 2 hours in 0.2 mL media. The spent media was removed, and cells were washed thrice with 1 X PBS before the addition of fresh media and irradiation with 980 nm continuous wave laser (CNI, Jilin, China) at 2.5 W cm^{-2} for 30 minutes. Cells were harvested with a scraper in 1 x Hanks balanced salt solution (HBSS), carboxy-H_2DCFDA (Molecular Probes, Eugene, OR, USA) was added at a working concentration of 5 µM and incubated for 10 minutes at room temperature. Flow cytometry was performed on BD FACS Canto to detect positive ROS levels using the FITC channel and Ce6 uptake using the APC channel. Cells without carboxy-H_2DCFDA added were used as a baseline to measure background fluorescence, and 1 x 10^4 cells per sample were acquired for analysis. The percentage of cells with positive ROS levels, and the geometric mean Ce6 fluorescence level data was obtained from the BD FACSDiva software. Overlay histogram profiles of all groups were created using flowing software (http://flowingsoftware.btk.fi/).

SOSG assay: UCNPs-Ce6 particles (0.5 mg mL^{-1}) solutions were prepared in a cuvette, and SOSG assay reagent (Molecular Probes, Eugene, OR, USA) was added to a working concentration of 10 µM. Irradiation of the solution with 980 nm continuous wave laser (CNI) was performed at 2.5 W cm^{-2}, and 0.1 mL of the solution was extracted at 5, 10, 15, 20, 30, 40, and 60 minutes to measure fluorescent SOSG at excitation/emission wavelengths of 490/525 nm with a microplate reader (Biotek, Synergy H1, Winooski, VT, USA).

Localization of Ce6 in mitochondria: MB49, RT4, T24, and UMUC3 cells (5 x 10^4) were plated in 8-well chamber slides in 0.2 mL volume overnight before treatment with free Ce6 or big UCNPs-Ce6 clusters containing 40 µM Ce6 concentration for 4 hours. A

solution (0.2 mL) containing Mitogreen (Abcam, Cambridge, UK) and Hoechst 33342 (Abcam) in the manufacturer's recommended concentrations was added to the cells and incubated for an additional 1 hour. The fluorescence was detected with an Olympus FV1000 TIRF confocal microscope (Tokyo, Japan) using LD 405 nm laser diode (Ce6 and Hoechst) and multi argon 488 nm laser (Mitogreen). Colocalization analysis of Ce6 and Mitogreen signals was performed using the Coloc 2 plugin available in Image J (Fiji).

Toxicity of UCNPs on urothelial carcinoma cell lines: UMUC3 and MB49 cells (1.5 x 10^4) were plated in 96 well plates overnight before treatment with various concentrations of big UCNPs-Ce6 clusters (0.1–1 mg mL^{-1}) with or without loaded Ce6 for 14 hours before MTS assay. Results were analyzed by referencing the percentage of cells in each treatment doses against control samples.

Visible light PDT efficacy of UCNPs-Ce6 particles in vitro: MB49 and RT4 cell lines (1.5 x 10^4) were plated in 96 well plates overnight and treatment with free Ce6 or UCNPs-Ce6 particles at various Ce6 concentrations (0.1–5 µM for MB49 and 5–20 µM for RT4) for 2 hours commenced the following day. Spheroids harvested at day 3 (300–500 µm diameter, 0.5 x 10^4 cells mL^{-1} seeding density) were used for efficacy assay with 3D organoids. Cells/ spheroids were washed twice with 1 x PBS and supplemented with fresh media (0.1 mL) before irradiation was performed with a visible light dual-LED device (405/660 nm) for 30 minutes in the dark. The device was powered with current from a DC power supply (Tektronix, PWS2326, Beaverton, OR, USA), and the current was measured with a multimeter (B&K Precision, 2709B, Yorba Linda, CA, USA) attached to the system via serial connection. The light dose applied is kept at 1 mW cm^{-2}. Cells were incubated at 37 °C for a further 24 hours before measuring monolayer cell viability with MTS assay (Promega, Madison, WI, USA) using 20 µL of MTS reagent.

Absorbance at 490 nm and 630 nm was measured after 4 hours with a microplate reader (Biotek). Results were analyzed by subtracting the OD 630 nm measurements from OD 490 nm raw data, and the percentage of cells in each sample group was referenced with their respective controls. Spheroid viability was measured by adding an equal volume of CellTiter-Glo® 3D reagent (Promega, Madison, WI, USA) to culture media for 25 minutes. Luminescence was recorded with a plate reader (Biotek) using an integration time of 1 second per well. The percentages of cells in each sample group were referenced with respective controls.

NIR light PDT efficacy of UCNPs-Ce6 particles in vitro: MB49 monolayer and 3D spheroids were prepared similarly to visible light PDT experiment. UCNPs-Ce6 particles were added at various concentrations (0.1–1 mg mL^{-1}) for 2 hours before cells/spheroids were washed thrice with 1 x PBS followed by irradiation with 980 nm continuous wave laser (CNI) at 2.5 W cm^{-2} for 30 minutes. An optical beam shutter attached to a shutter controller (Thor Labs, New Jersey, USA) programmed to open for 0.1 seconds and close for 0.9 seconds was used to prevent overheating of the cells and spheroids. The cells/spheroids were further incubated for 24 hours before viability assay was performed with MTS or CellTiter-Glo® 3D reagent.

In vivo dark toxicity analysis in mice: Female C57BL/6J mice (aged 4-6 weeks old) were purchased from InVivos, Singapore. The animal protocol was approved by the Institutional Animal Care and Use Committee (IACUC), National University of Singapore (NUS). The mice underwent an acclimatization period of 1 week at the NUS Comparative Medicine facility before the commencement of the experiment. Mice were anesthetized via intraperitoneal administration of ketamine (75mg kg^{-1}) and medetomidine (1mg kg^{-1}) before implantation and treatment procedures. Subcutaneous tumors were induced in mice by implanting 3×10^6 MB49-PSA bladder cancer cells in

the lower flank of each mice. Tumor growth was monitored daily, and visible tumor size was measured using a caliper and a ruler. Tumor volume was calculated using the formula: $v = \frac{(ab^2)}{2}$ where a is the longest tumor dimension, and b is the perpendicular width of a. Treatment started at Day 6 post-implantation when the tumor volume exceeded 50 mm^3. Mice were randomly divided into control saline (B.Braun, Melsungen, Germany) and small UCNPs-Ce6 clusters treatment groups. Small UCNPs-Ce6 clusters treatment was given at 10 mg kg^{-1} body weight, and the clusters were prepared in a 2 mg mL^{-1} concentration resuspended in normal saline. Intratumoral administration of 0.1 mL saline or approximately 0.1 mL of small UCNPs-Ce6 clusters was administered to the mice according to individual body mass. The treatment solution was injected in three different sites within the tumor mass to ensure even distribution of saline/small clusters. Intraperitoneal atipamezole reversal at 1 mg kg^{-1} body weight was given to mice after the procedures were completed. Treatment with saline or small UCNPs-Ce6 clusters was repeated on Day 12, and mice were terminated on Day 18. The body weight and tumor volume were recorded throughout the entire experiment (once every two days) to monitor their well-being. Mice were terminated if they had lost 20 % body weight, or the tumor growth had reached the ethical limit of 750 mm^3. Mice organs (liver, spleen, kidneys, lungs, and heart) were harvested after termination, and each organ mass was recorded. Tumor size data were normalized against each respective mouse tumor volume at the start of the experiment (Day6) over time.

Statistical Analysis: All statistical analysis was performed using GraphPad v6 software (GraphPad, SanDiego, CA, USA). Comparisons of means between multiple groups were performed using one-way ANOVA with a Bonferroni post-hoc test. Mann-Whitney independent sample test was used to compare between two groups due to small sample sizes. Differences between means were considered statistically significant when the two-

tailed test *p*-value < 0.05. Representation of the *p*-value was indicated in each respective figure. At least 100 objects were analyzed for each sample for the statistical analysis of fractions of different particles for different polymer/UCNPs weight ratios (Figure 1n).

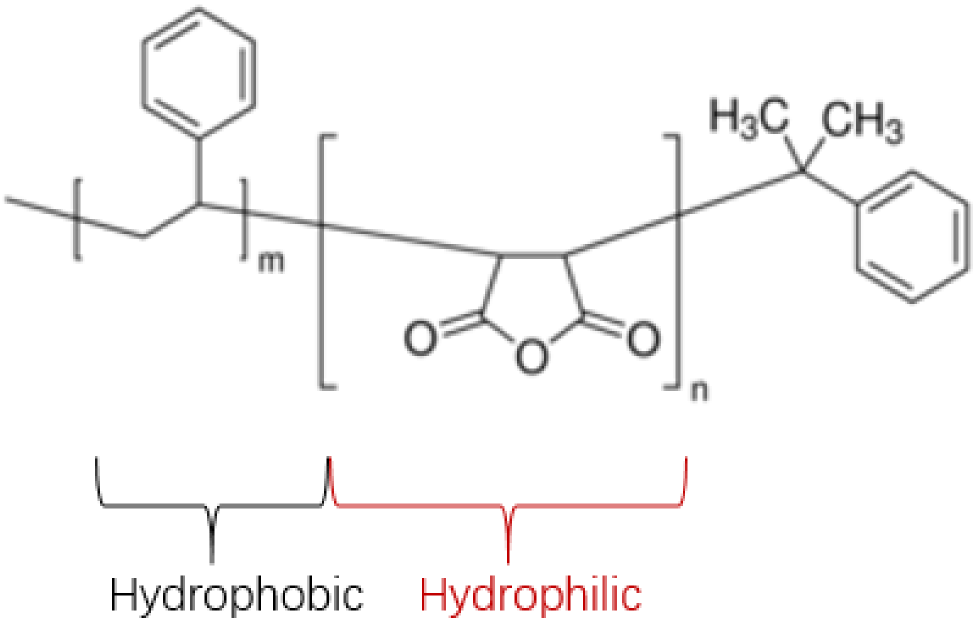

Figure S1. The chemical structure of PSMA.

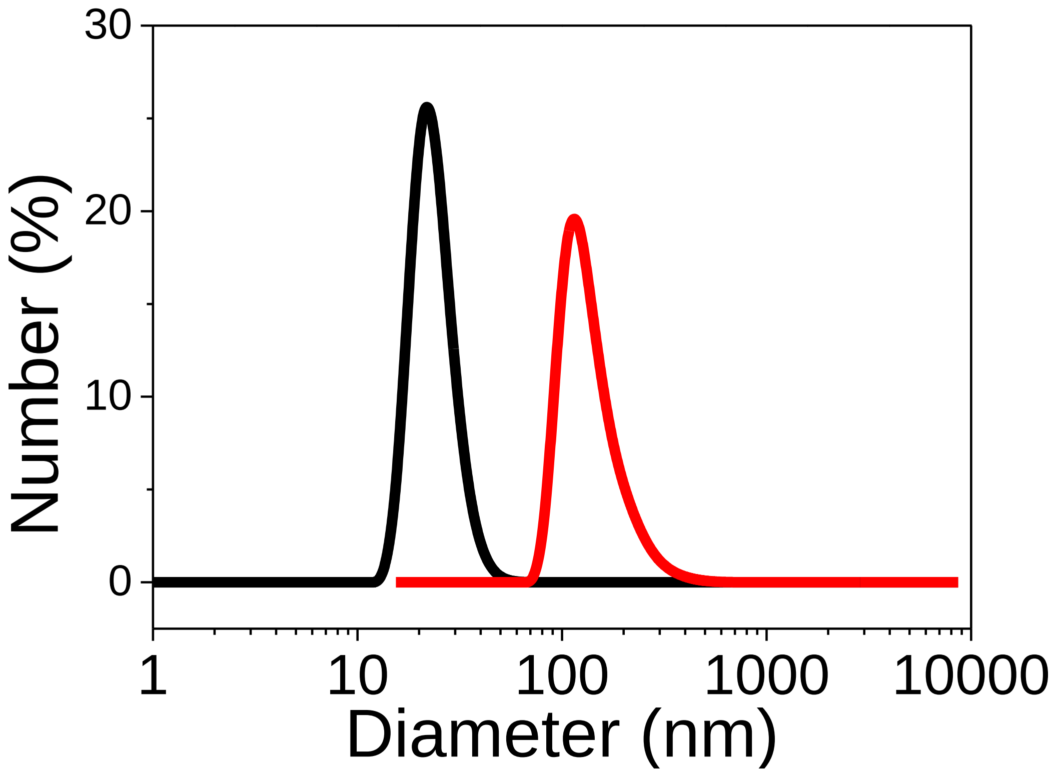

Figure S2. The DLS results of UCNPs in THF (black line) and UCNPs clusters (red line) in water.

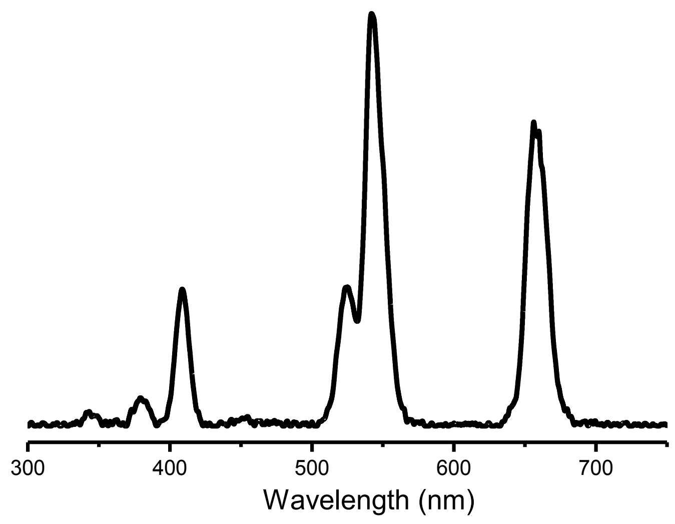

Figure S3. 980 nm laser-excited upconversion emission spectra of UCNPs in cyclohexane solution at room temperature.

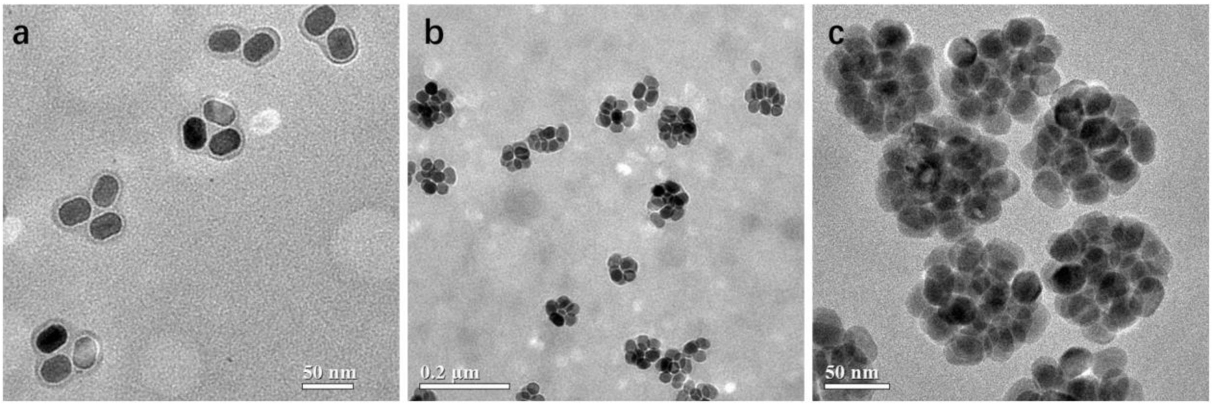

Figure S4. Representative TEM micrographs of UCNPs morphology in water after the THF evaporation with different polymer/UCNPs weight ratios: a) 8.75; b) 17.50; c) 26.25. UCNPs: $NaYF_4$: 20%Yb, 2%Er.

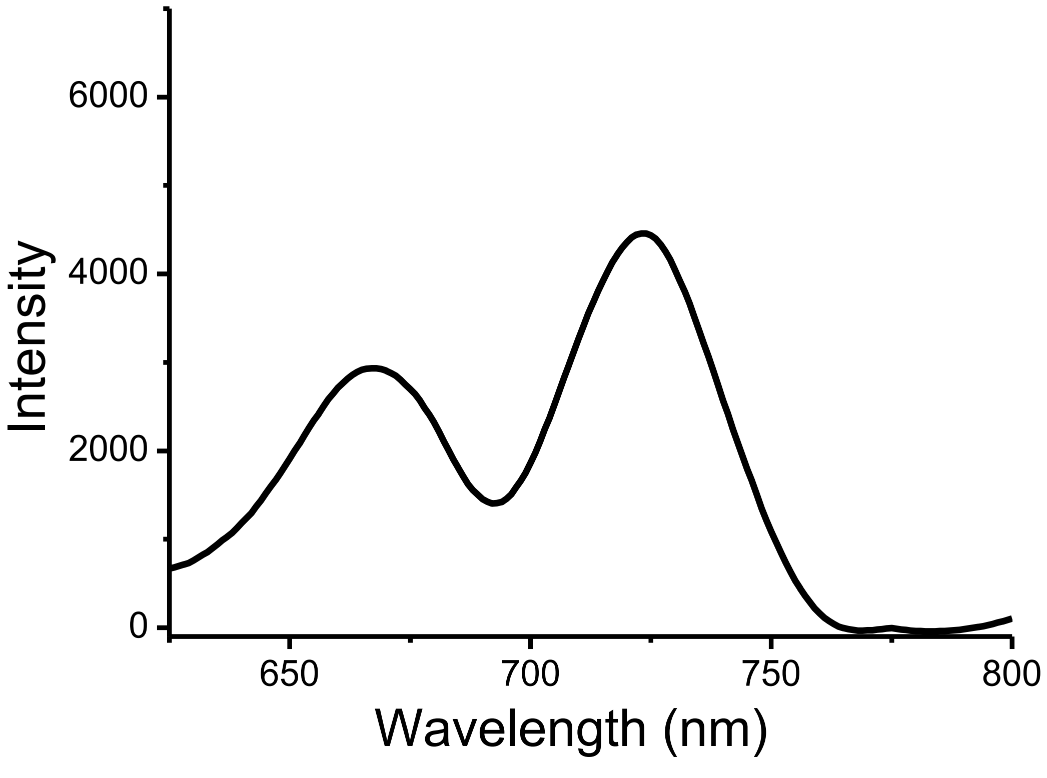

Figure S5. The emission spectrum of UCNPs clusters containing Ce6 under 365 nm.

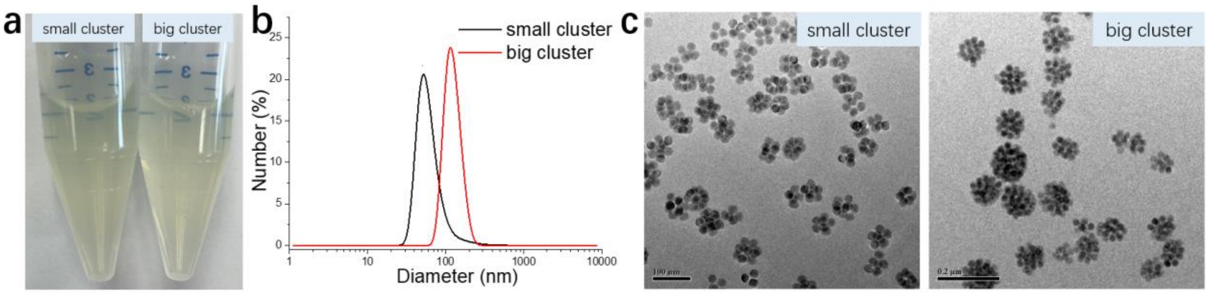

Figure S6. Stability test for the small and big UCNPs clusters loaded with Ce6 after 140 days at 4 ^{o}C. Photograph image a), DLS b), and TEM images (c) of the small and big UCNPs clusters. From the pictures above, the small and big UCNPs clusters solutions remained clear. The DLS results showed a narrow size diffusion, and form the TEM images, functional clusters morphology could be confirmed. In summary, the UCNPs clusters were stable even after 140 days of storage.

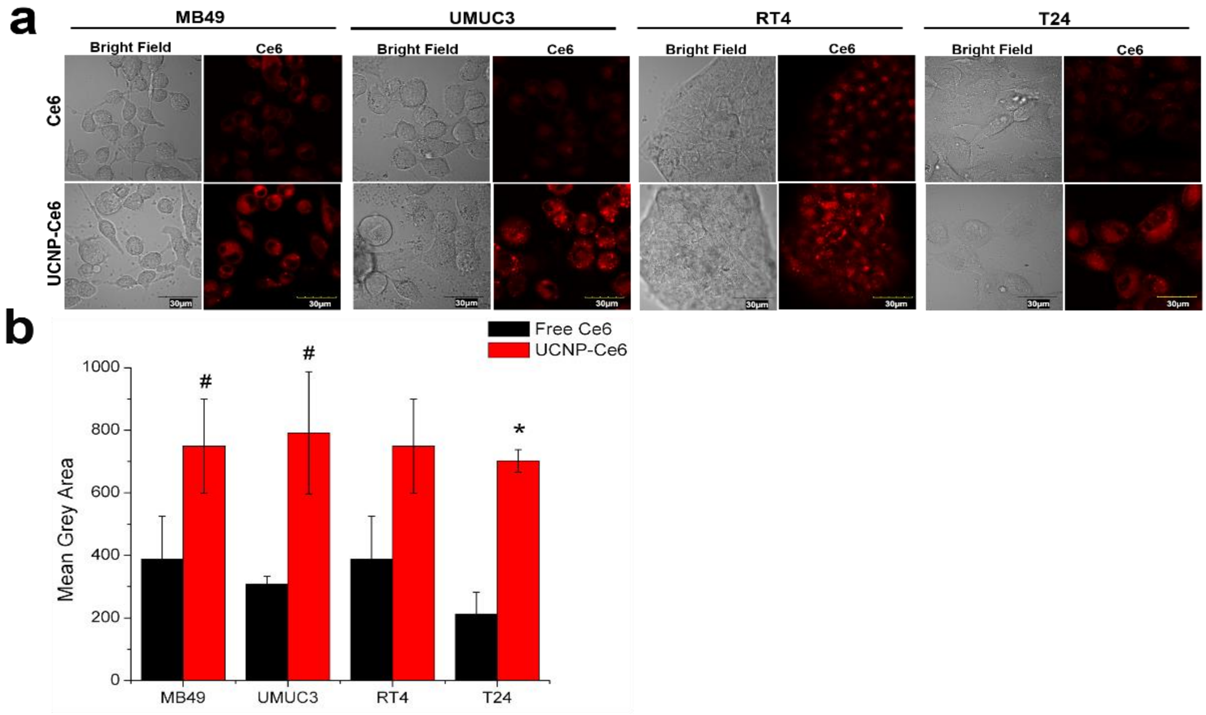

Figure S7. Ce6 uptake in bladder cancer cell lines. a) Comparisons of uptake between free Ce6 and big UCNPs-Ce6 clusters. Cells were treated with 20 μM Ce6 for 14 hours

and viewed under an Olympus FV1000 TIRF confocal microscope to detect Ce6 fluorescence. Images were obtained under 60x magnification with 2x digital zoom. Scale bar, 30 µm. b) Images were obtained from 3 random spots in each sample. Ce6 fluorescence intensity was analyzed using Fiji (Image J) software. Regions of interests were selected, and the mean grey area values were plotted for fluorescence intensity comparisons. Data were represented as mean ± SD. This experiment was performed once with three distinct samples (n=3). #, * denotes significance to free Ce6. # p< 0.05, *p< 0.005. Mann-Whitney independent sample test was used to compare between two groups.

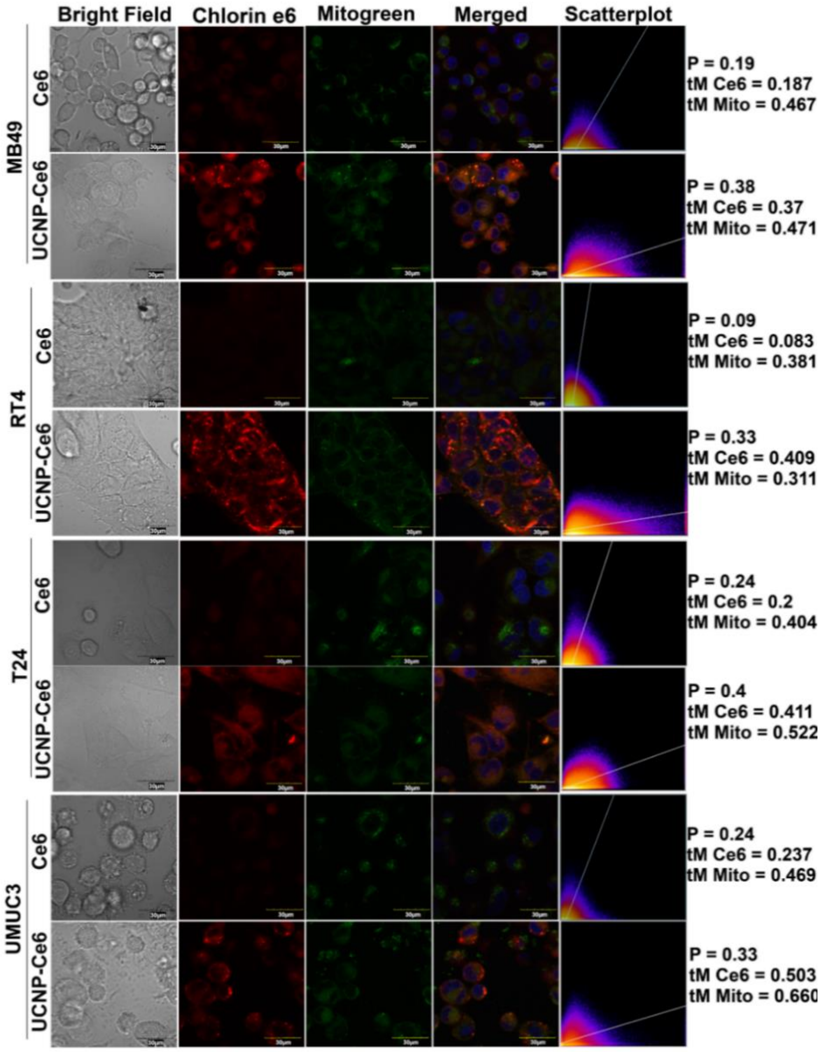

Figure S8. Subcellular localization of Ce6 in bladder cancer cell lines. Cells were treated with 40 µM free Ce6 or big clusters containing 40 µM Ce6 for 4 hours and subsequently stained with Mitogreen and Hoechst 33342 nuclear stain for 1 hour at 37 °C. The samples were viewed using an Olympus FV1000 TIRF confocal microscope to detect the distribution of Ce6 (red), mitochondria (green), and nuclear staining (blue). Images were obtained under 60x magnification with 2x digital zoom. Colocalization analysis was performed using the Coloc 2 plugin in Image J (Fiji). Scale bar, 30 µm. P = Pearson's correlation coefficient, tM = thresholded Mander's split colocalization coefficient. This experiment was performed twice in the MB49 cell line and once in the RT4, T24, and UMUC3 cell lines.

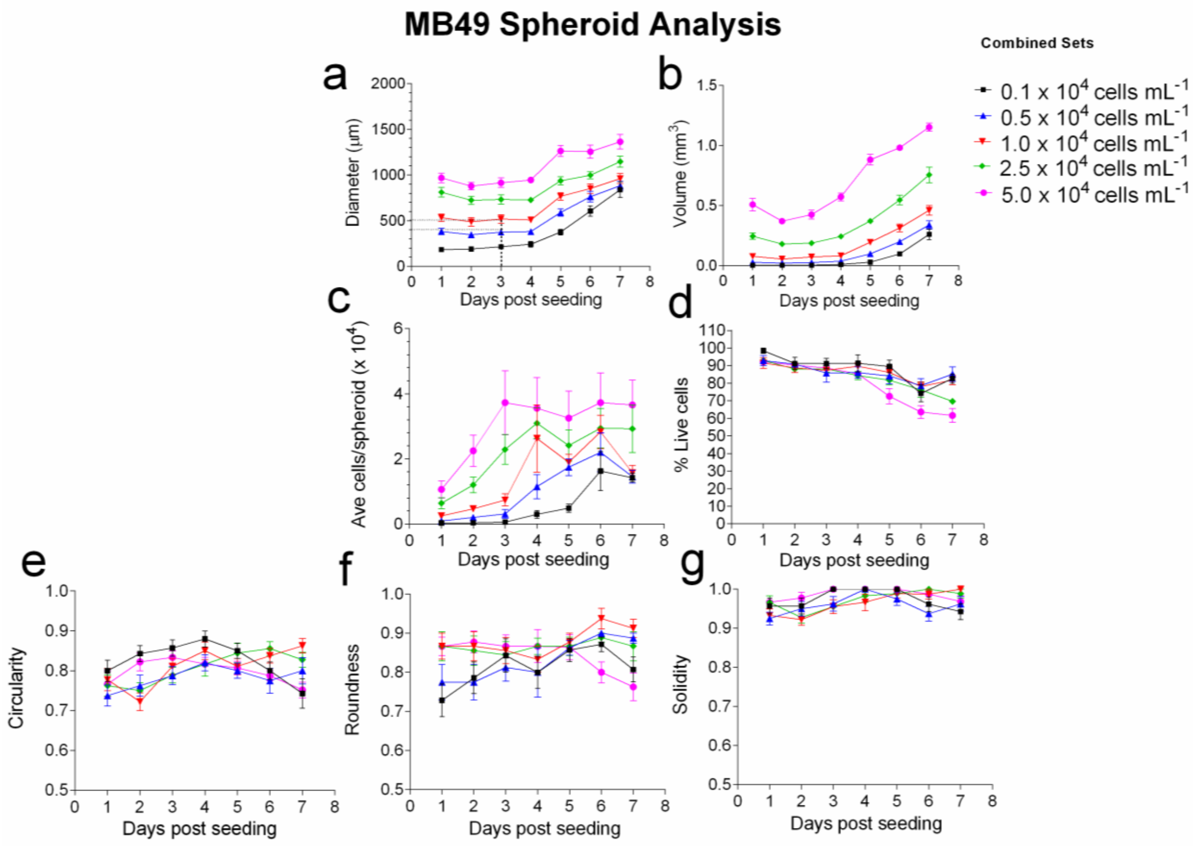

Figure S9. Characteristics of MB49 spheroids in culture over time. a) Diameter (min Feret). b) Volume. c) The average number of cells per spheroid. d) Percentage of live cells. e) Circularity. f) Roundness and g) solidity. Data were represented as mean ± SEM

(n=9) with the exclusion of the average number of cells per spheroid and the percentage of live cells (n=4).

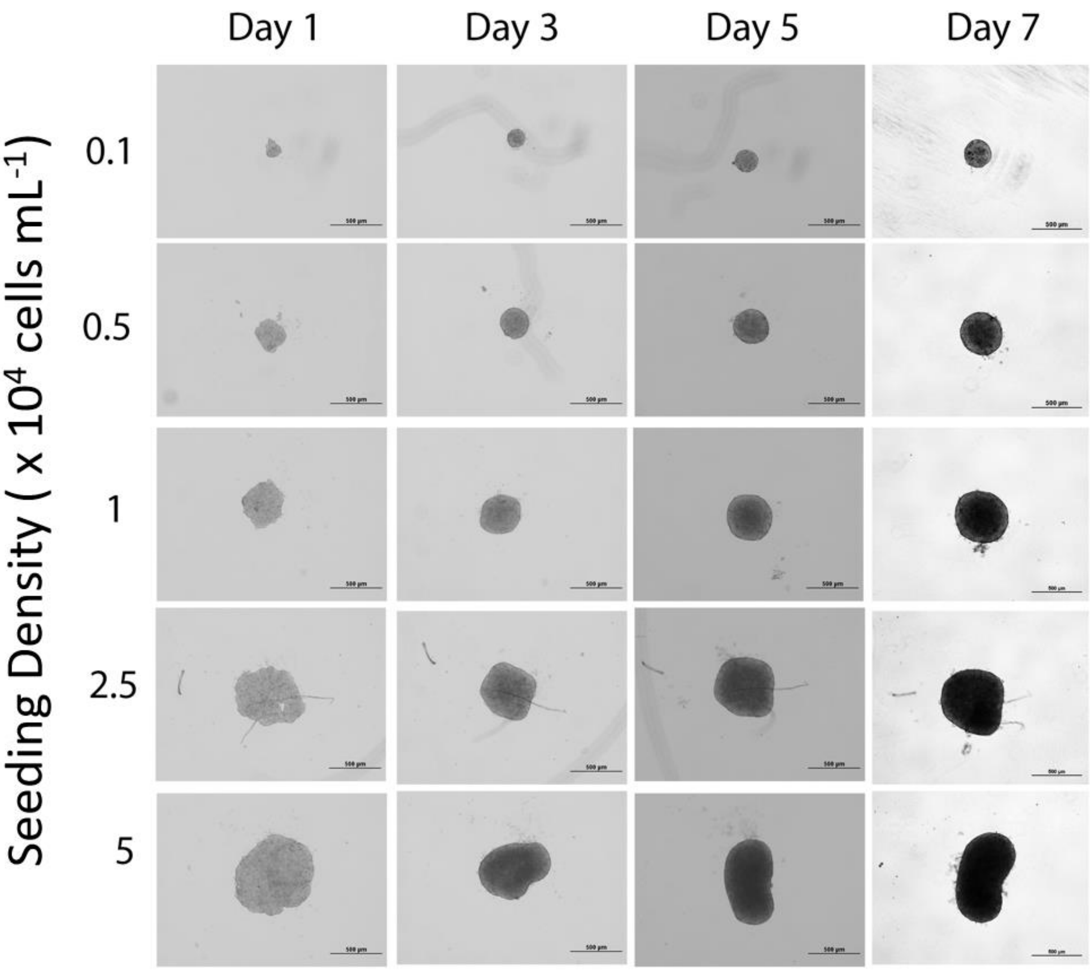

Figure S10. Characterization of RT4 spheroids. Phase-contrast images of RT4 spheroid growth for seven days in culture with different seeding concentration (cells mL^{-1}, 0.2 mL plated each well). Images were obtained at 4x objective. Scale bar, 500 µm.

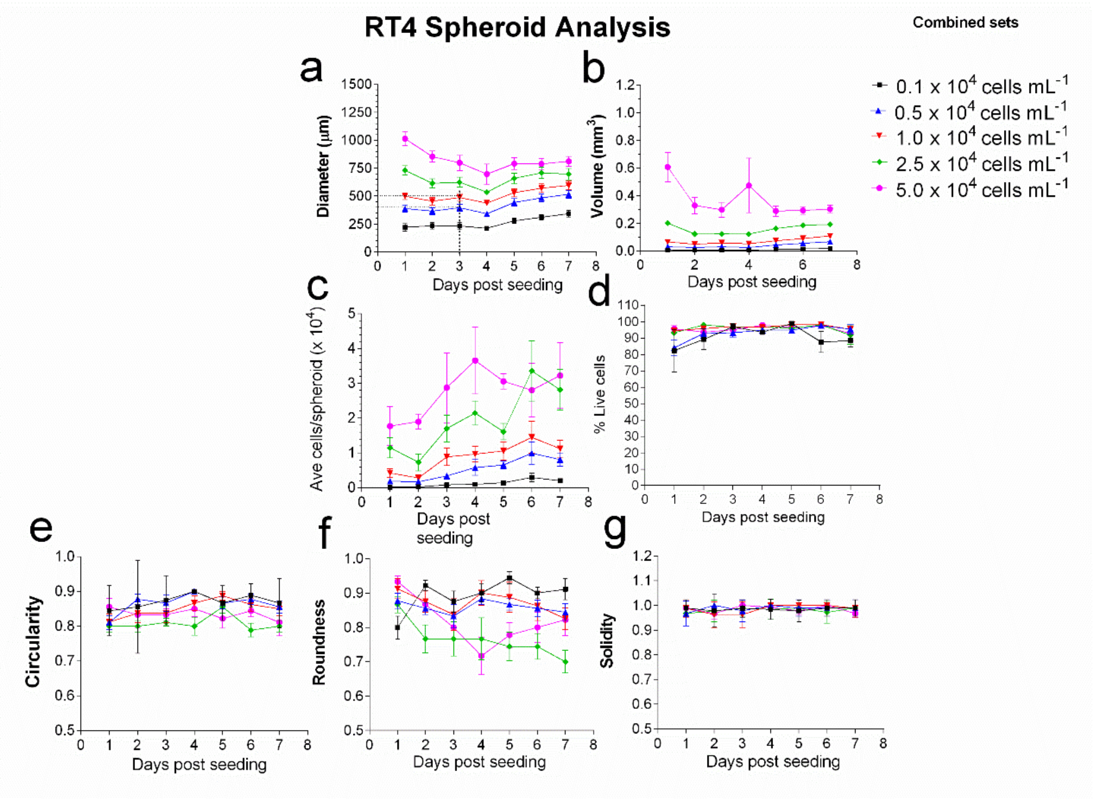

Figure S11. Characteristics of RT4 spheroids in culture over time. a) Diameter (min Feret). b) Volume. c) The average number of cells per spheroid. d) Percentage of live cells. e) Circularity. f) Roundness and g) solidity. Data were represented as mean ± SEM (n=9) with the exclusion of the average number of cells per spheroid and the percentage of live cells (n=5).

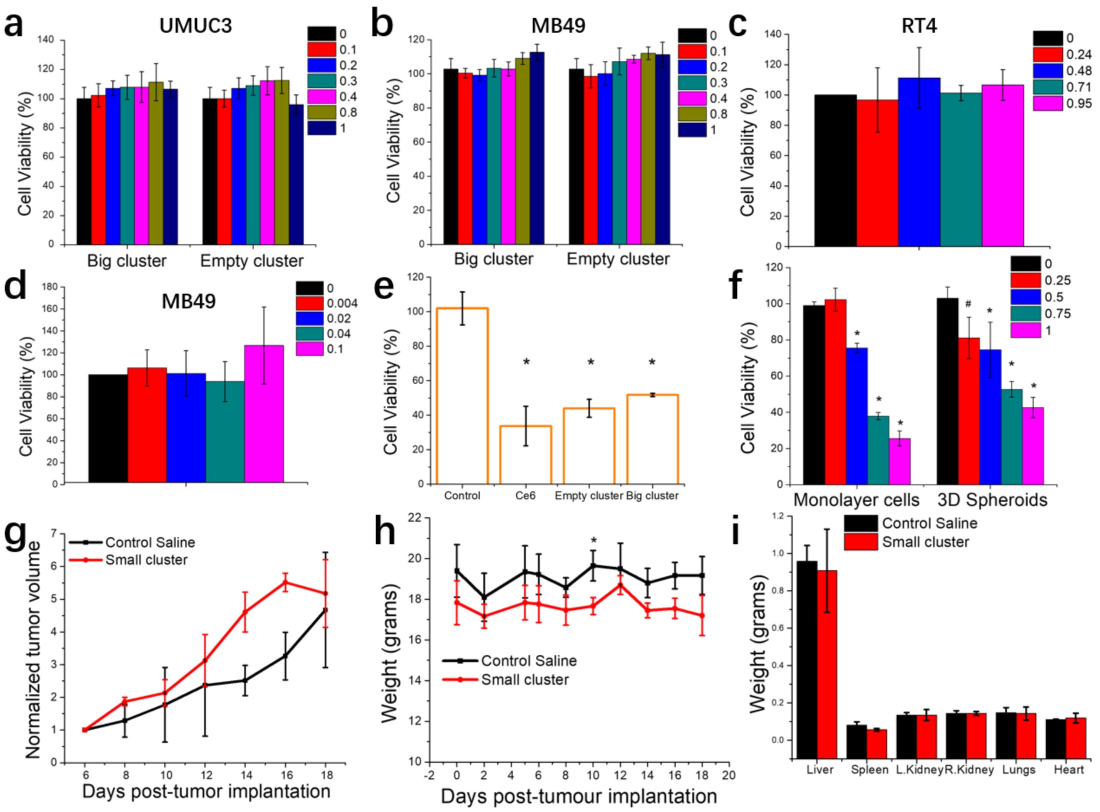

Figure S12. Dark and phototoxicity of UCNPs clusters. Dark toxicity of 14 hours overnight incubation with various concentrations of big UCNPs clusters on a) UMUC3 and b) MB49 monolayer cell lines. Cells were incubated with UCNPs clusters for 14 hours before the MTS assay. This experiment was performed twice in triplicates (n=6). Irradiated toxicity of empty UCNPs clusters on c) RT4 and d) MB49 monolayer cell line. Cells were treated for 2 hours with varying concentrations of empty big UCNPs clusters (devoid of Ce6) that was used for the PDT experiment before removal and washings followed by irradiation with dual light LED (405/660 nm). A further 24 hours of incubation was done before the MTS assay. This experiment was performed thrice in duplicates (n=6). All UCNPs clusters concentrations are in mg mL^{-1}. e) Dark toxicity of high clusters and Ce6 concentration treatments. MB49 cells were treated with 1.7 mg mL^{-1} big UCNPs clusters or 40 μM free Ce6 for 4 hours before removal of UCNPs clusters/Ce6 containing media and washings. Cells were further incubated for 24 hours before the MTS assay was performed. This experiment was performed once in triplicates

(n=3). * denotes significant compared to control $p< 0.005$. f) Dark toxicity of UCNPs-Ce6 small clusters concentration treatments in monolayer cells and 3D spheroids. MB49 monolayer cells or 3D spheroids were treated with various concentrations of small clusters for 2 hours before the removal of UCNPs-Ce6 clusters containing media followed by washings. Cells were further incubated for 24 hours before viability assays. The experiment was performed twice in duplicates (n=4). #,* denotes significant compared to control # $p< 0.05$, * $p<0.005$. *In vivo* subcutaneous tumor dark toxicity study in mice. g) Normalized tumor volume as a function of time during the monitoring period. Treatments were administered on Day 6 and Day 12. h) Bodyweight monitoring over the treatment period. * denotes a significant difference between the two treatment groups ($p< 0.05$). (i) Comparisons of organ weights between control saline and small UCNPs-Ce6 clusters treated mice. The *in vivo* experiment was performed twice in duplicates (n=4). All data in this figure were represented as mean ± SD. Concentration legends are in mg mL^{-1}. One-way ANOVA with a Bonferroni post-hoc analysis was used for comparison among multiple groups. Mann-Whitney independent sample test was used to compare between two groups.

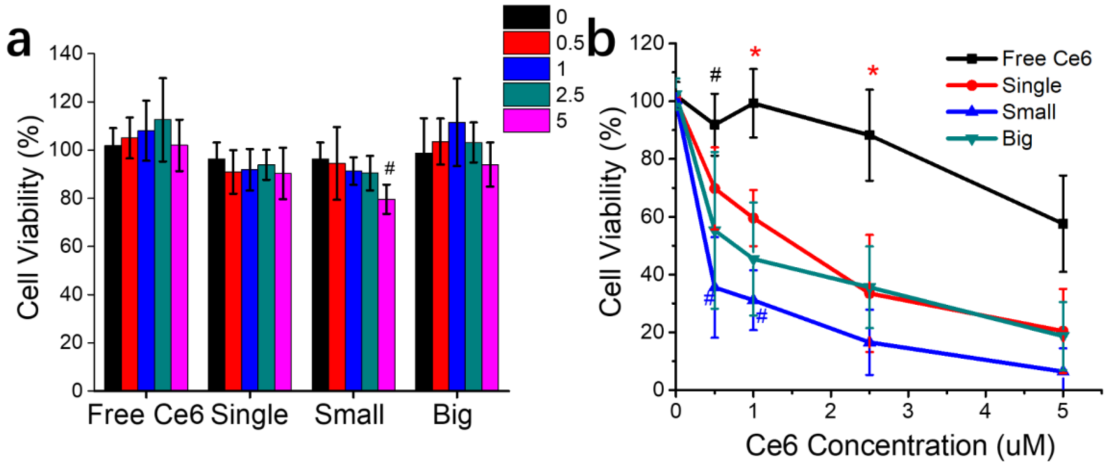

Figure S13. Dark toxicity assay was performed for 2 hours on (a) MB49 spheroids (# denotes significance compared to control). Concentration legends denote Ce6 concentrations (See Table S2 for respective cluster concentrations). *In vitro* visible light PDT efficacy of UCNPs-Ce6 clusters formulations in MB49 3D (b) spheroids (black # denotes significance compared to small and big groups, blue # denotes significance compared to single groups, and red * denotes significance compared to single, small and big groups). Treatment with UCNPs-Ce6 formulations for 2 hours was done, followed by irradiation with a dual-LED device (405/660 nm) for 30 minutes. Data were represented as mean ± SD. The experiment was performed thrice in duplicates (n=6). #p <0.05, *p$<$ 0.005. One-way ANOVA with a Bonferroni post-hoc analysis was used for comparison among multiple groups.

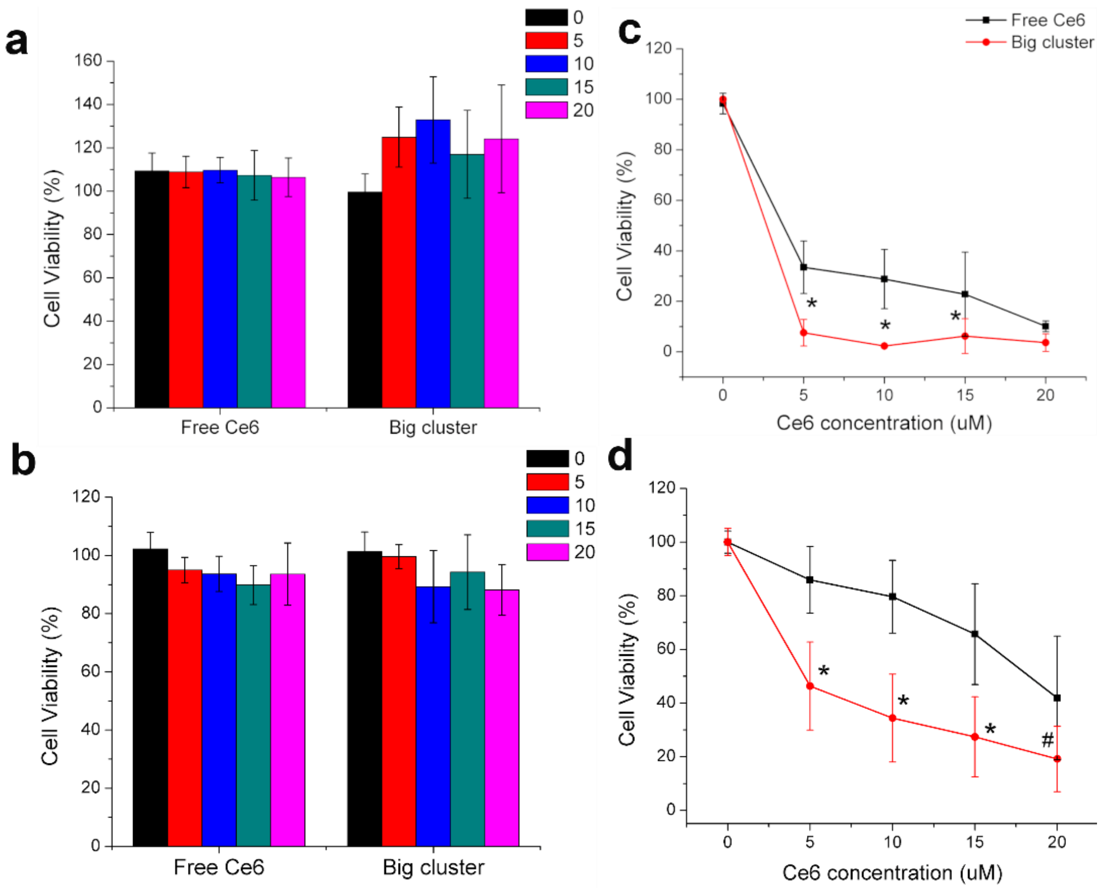

Figure S14. *In vitro* PDT efficacy of big UCNPs-Ce6 clusters. Dark toxicity assay was performed for 2 hours on a) RT4 monolayer and b) RT4 spheroids. Concentration legends denote Ce6 concentrations. (See Figure S12c for respective cluster concentrations). *In vitro* visible light PDT efficacy of big UCNPs-Ce6 clusters formulations in c) RT4 monolayer cell line and d) RT4 3D spheroids. Treatment with free Ce6 or big UCNPs-Ce6 formulations for 2 hours was done, followed by irradiation with a dual-LED device (405/660 nm) for 30 minutes. Data were represented as mean ± SD. This experiment was performed thrice in duplicates (n=6). #p <0.05, *p< 0.005. One-way ANOVA with a Bonferroni post-hoc analysis was used for comparison among multiple groups for the dark toxicity experiment. Mann-Whitney independent sample test was used to compare between free Ce6 and big cluster groups.

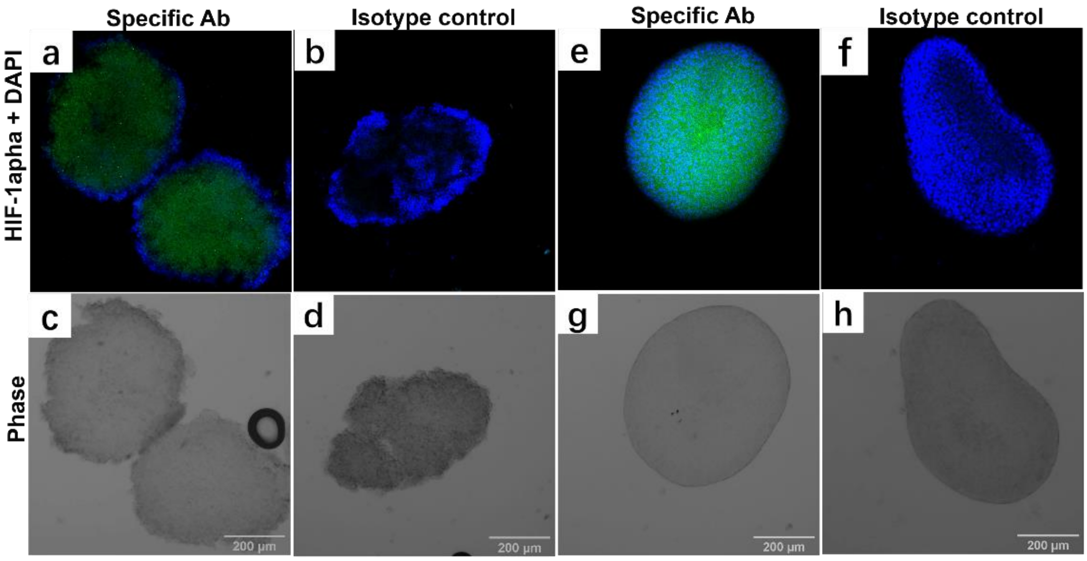

Figure S15. Detection of hypoxia in MB49 and RT4 spheroid core. Spheroids were immuno-labeled with HIF-1α specific antibodies (green) followed by nuclear staining with DAPI (blue). a) MB49 and e) RT4 spheroids labeled with HIF-1α antibodies. b) MB49 and f) RT4 spheroids labeled with isotype control. c, d, g, h Respective phase-contrast images. Images were obtained with a Zeiss LSM710 confocal microscope with a 10x objective lens. Scale bar, 200 um. Green: HIF-1alpha, Blue: DAPI.

Table S1. Characteristics of Day 3 spheroids at 600-800 µm diameter size (used for Ce6 penetration studies).

	RT4	MB49
Seeding density	2.5 x 10^4 cells mL^{-1}	2.5 x 10^4 cells mL^{-1}
Diameter	624.1 ± 134	732.8 ± 152
% increase cell number from Day 1-Day 3	147	344
Viability (%)	96.6 ± 2.1	86.8 ± 5.0
Circularity	0.811 ± 0.03	0.789 ± 0.06
Roundness	0.767 ± 0.15	0.844 ± 0.1
Solidity	0.962 ± 0.05	0.956 ± 0.05

Data were represented as mean ± SD.

Table S2. The respective concentration of clusters used for each Ce6 concentrations used in intracellular uptake/penetration and visible light PDT studies.

	UCNPs concentration (mg mL^{-1})		
Ce6 concentration	Single	Small	Big
0.1	0.0047	0.00348	0.0029
0.5	0.0235	0.0174	0.0145
1	0.047	0.0348	0.029
2.5	0.1175	0.087	0.0725
5	0.235	0.174	0.145

Table S3. Characteristics of Day 3 spheroids at 400-500 µm diameter size (used for PDT efficacy studies).

	RT4	MB49
Seeding density	0.5 x 10^4 cells mL^{-1}	0.5 x 10^4 cells mL^{-1}
Diameter	396 ± 94.9	374 ± 93.3
% increase cell number from Day 1-Day 3	82.7	374.4
Viability (%)	93.2 ± 5.8	91.65 ± 5.3
Circularity	0.867 ± 0.071	0.786 ± 0.064
Roundness	0.833 ± 0.1	0.813 ± 0.1

Data were represented as mean ± SD.

Table S4. IC50 values of MB49 visible light PDT with free Ce6 and UCNPs clusters.

	IC50 values (µM)	
	Monolayer	3D spheroids
Free Ce6	2	-
Single UCNPs conjugated Ce6	1.7	1.6
Small UCNPs-Ce6 clusters	1.2	0.2
Big UCNPs-Ce6 clusters	1	0.8

Table S5. IC50 values of RT4 visible light PDT with free Ce6 and UCNPs clusters.

	IC50 values (μM)	
	Monolayer	3D spheroids
Free Ce6	3.5	18
Big UCNPs-Ce6 clusters	2.6	4.8

Table S6. The respective concentration of Ce6 for each UCNPs concentrations used in ROS induction, NIR PDT, and *in vivo* dark toxicity studies.

	Ce6 concentration (μM)		
UCNPs concentration (mg mL^{-1})	Single	Small	Big
0.1	2	2.4	2.1
0.25	5	6	5.25
0.5	10	12	10.5
0.75	15	18	15.75
1	20	24	21

Table S7. Effects of small UCNPs-Ce6 clusters treatment on monolayer MB49 cell line in combination with (or without) NIR PDT.

UCNPs clusters concentration ($mg\ mL^{-1}$)	Irradiation (Cell Viability, %)	No irradiation (Cell Viability, %)	*p*-value
0	106.2 ± 18.5	99.0 ± 2.1	0.0892
0.1	96.5 ± 9.3	90.8 ± 3.3	0.035 *
0.25	88.1 ± 16.2	102.3 ± 6.3	0.197
0.5	44.6 ± 7.2	75.6 ± 2.6	0.0043 *
0.75	17.4 ± 0.14	37.9 ± 2.0	< 0.0001 *
1	11.9 ± 2.4	25.5 ± 4.2	0.1333

Data were represented as mean ± SD. * denotes significant difference between irradiated and non-irradiated groups. Mann-Whitney independent sample test was used to compare between two groups.

Table S8. Effects of small UCNPs-Ce6 clusters treatment on MB49 3D spheroids in combination with (or without) NIR PDT.

UCNPs clusters concentration ($mg\ mL^{-1}$)	Irradiation (Cell Viability, %)	No irradiation (Cell Viability, %)	*p*-value
0	102.8 ± 9.7	103.1 ± 6.2	0.6286
0.25	65 ± 5.3	81.1 ± 11.4	0.0286 *
0.5	62.7 ± 5.6	74.5 ± 15.3	0.3429
0.75	33.7 ± 3.8	52.7 ± 4.3	0.0006 *
1	16.1 ± 6.5	42.6 ± 5.6	0.0286 *

Data were represented as mean ± SD. * denotes significant difference between irradiated and non-irradiated groups. Mann-Whitney independent sample test was used to compare between two groups.
